# Dialects of the DNA Uptake Sequence in *Neisseriaceae*

**DOI:** 10.1371/journal.pgen.1003458

**Published:** 2013-04-18

**Authors:** Stephan A. Frye, Mariann Nilsen, Tone Tønjum, Ole Herman Ambur

**Affiliations:** 1 Centre for Molecular Biology and Neuroscience (CMBN), University of Oslo and Oslo University Hospital, Rikshospitalet, Oslo, Norway; 2 Department of Microbiology, University of Oslo and Oslo University Hospital, Rikshospitalet, Oslo, Norway; University of Geneva Medical School, Switzerland

## Abstract

In all sexual organisms, adaptations exist that secure the safe reassortment of homologous alleles and prevent the intrusion of potentially hazardous alien DNA. Some bacteria engage in a simple form of sex known as transformation. In the human pathogen *Neisseria meningitidis* and in related bacterial species, transformation by exogenous DNA is regulated by the presence of a specific DNA Uptake Sequence (DUS), which is present in thousands of copies in the respective genomes. DUS affects transformation by limiting DNA uptake and recombination in favour of homologous DNA. The specific mechanisms of DUS–dependent genetic transformation have remained elusive. Bioinformatic analyses of family *Neisseriaceae* genomes reveal eight distinct variants of DUS. These variants are here termed DUS dialects, and their effect on interspecies commutation is demonstrated. Each of the DUS dialects is remarkably conserved within each species and is distributed consistent with a robust *Neisseriaceae* phylogeny based on core genome sequences. The impact of individual single nucleotide transversions in DUS on meningococcal transformation and on DNA binding and uptake is analysed. The results show that a DUS core 5′-CTG-3′ is required for transformation and that transversions in this core reduce DNA uptake more than two orders of magnitude although the level of DNA binding remains less affected. Distinct DUS dialects are efficient barriers to interspecies recombination in *N. meningitidis*, *N. elongata*, *Kingella denitrificans*, and *Eikenella corrodens*, despite the presence of the core sequence. The degree of similarity between the DUS dialect of the recipient species and the donor DNA directly correlates with the level of transformation and DNA binding and uptake. Finally, DUS–dependent transformation is documented in the genera *Eikenella* and *Kingella* for the first time. The results presented here advance our understanding of the function and evolution of DUS and genetic transformation in bacteria, and define the phylogenetic relationships within the *Neisseriaceae* family.

## Author Summary

Through computational and biological methods, this work analyzes the function and evolution of short DNA sequences called DNA uptake sequences (DUS) that regulate genetic transformation of bacteria in the family *Neisseriaceae*. Previous studies show that DUS affects transformation favourably. Here, for the first time, we document the existence of eight distinct DUS dialects that display differences in their respective nucleotide sequence that limits genetic “communication” between species. This suggests that each DUS dialect represents a barrier to horizontal gene transfer of heterologous DNA contributing to genetic isolation. Single nucleotide analysis of DUS was used to identify a three nucleotide core sequence common to all DUS dialects and essential for transformation. The discovery of multiple DUS dialects emphasizes that homologous recombination, allelic reassortment, and bacterial “sex” play an important role in the evolutionary past of *Neisseriaceae* species.

## Introduction

Transformation in bacteria is a complex process involving uptake of naked extracellular DNA followed by homologous recombination (HR). Different reproductive barriers have evolved in diverse transformation-competent bacteria, which distinguish in favour of acquisition and recombination of homologous DNA sequences and discriminate against heterologous and potentially hazardous DNA [1]. In particular, interspecies recombination with heterologous DNA in single cellular organisms could cause gene disruptions and/or disturb sensitive cellular processes, which could in turn have adverse phenotypic consequences. Adaptations that may contribute to sexual isolation and at the same time promote genetic stability include restriction modification systems, fratricide in streptococci and cannibalism in *Bacillus subtilis*, quorum-sensing, biofilm formation and HR regulation and suppression [2], [3], [4], [5]. Transformation in *Neisseria* sp. and members of the *Pasteurellaceae* family is unique in the requirement for short uptake sequences in the transforming DNA, named DNA Uptake Sequences (DUS) and Uptake Signal Sequences (USS), respectively [6], [7]. The genomes of these organisms harbour thousands of DUS and USS, constituting up to 1% of their entire chromosomes [8], [9], [10]. DUS has accumulated in the core genome, *i.e.* the set of common genes, of *N. meningitidis*, *N. gonorrhoeae* and *N. lactamica* and was found to maintain its sequence identity from frequent recombination [11]. DUS was first identified in *N. gonorrhoeae* as a 10-mer (5′-GCCGTCTGAA-3′) and has been documented functional in transformation of meningococci and gonococci [7], [12], [13]. Later, a revised 12-mer DUS (5′-AT-GCCGTCTGAA-3′, here named AT-DUS) was shown to elevate transformation further [14], [15]. High level expression of the competence and minor pilin protein ComP has been shown to increase DUS-specific uptake, and a definite association between DUS and ComP was published recently [16], [17], [18]. A linear relationship between the number of DUS and the ability to competitively inhibit the uptake of radio-labelled DNA in *N. gonorrhoeae* has been documented, suggesting initial surface binding of DUS [19]. An additive effect of DUS has been documented also in transformation experiments in *N. meningitidis*, although no linear relationship between the number of DUS and transformation frequencies was evident [20]. Importantly, DNA binding and uptake assays do not fully correlate with the outcome of transformation assays, indicating that more than one level of DUS specificity exist [15]. Recently an influence of DUS location relative to homologous and recombinogenic regions of transforming DNA was demonstrated, suggesting that DUS may initiate DNA processing by a yet undefined way [20]. Two versions of USS have been described in *Pasteurellaceae*: version A (5′-AAGTGCGGT-3′), named Hin-USS, is found in *Haemophilus influenzae* and *Actinobacillus actinomycetemcomitans* (now named *Aggregatibacter actinomycetemcomitans*) and USS version B (5′-ACAAGCGGT-3′), named the Apl-USS subtype, is found in *Actinobacillus pleuropneumoniae* [21]. DUS-like repeat sequences have been described for *N. subflava* and *N. sicca* [22] and recently also in *N. elongata* [23]. Different variants of DUS are here termed DUS dialects alluding to their role as nucleotide ‘words’ in genetic ‘communication’ and in concordance with the previous use of the term ‘dialects’ in genetic contexts [24].

Even though DUS seems to have disseminated in the genus *Neisseria*, virtually nothing is known about DUS repeats in the family *Neisseriaceae* genera *Kingella*, *Eikenella* and *Simonsiella*. To fill this knowledge gap, the work presented here examines DUS specificity and dialects within the family *Neisseriaceae* [11], [25]. The results reveal the presence of eight DUS dialects in different branches of the robust *Neisseriaceae* phylogenetic tree. In transformation assays, the DUS sequence divergence negatively influences inter-species transfer of DNA. A DUS core of only three nucleotides is present in all dialects and is strictly required for transformation. Assays with radiolabelled DNA show species specific relevance of DUS dialects for both binding and uptake of DNA. This work supports the idea that DUS specificity is a highly efficient barrier to interspecies transformation, that has great impact on the evolution of the *Neisseriaceae*.

## Results

### DUS dialects

**Figure 1:**
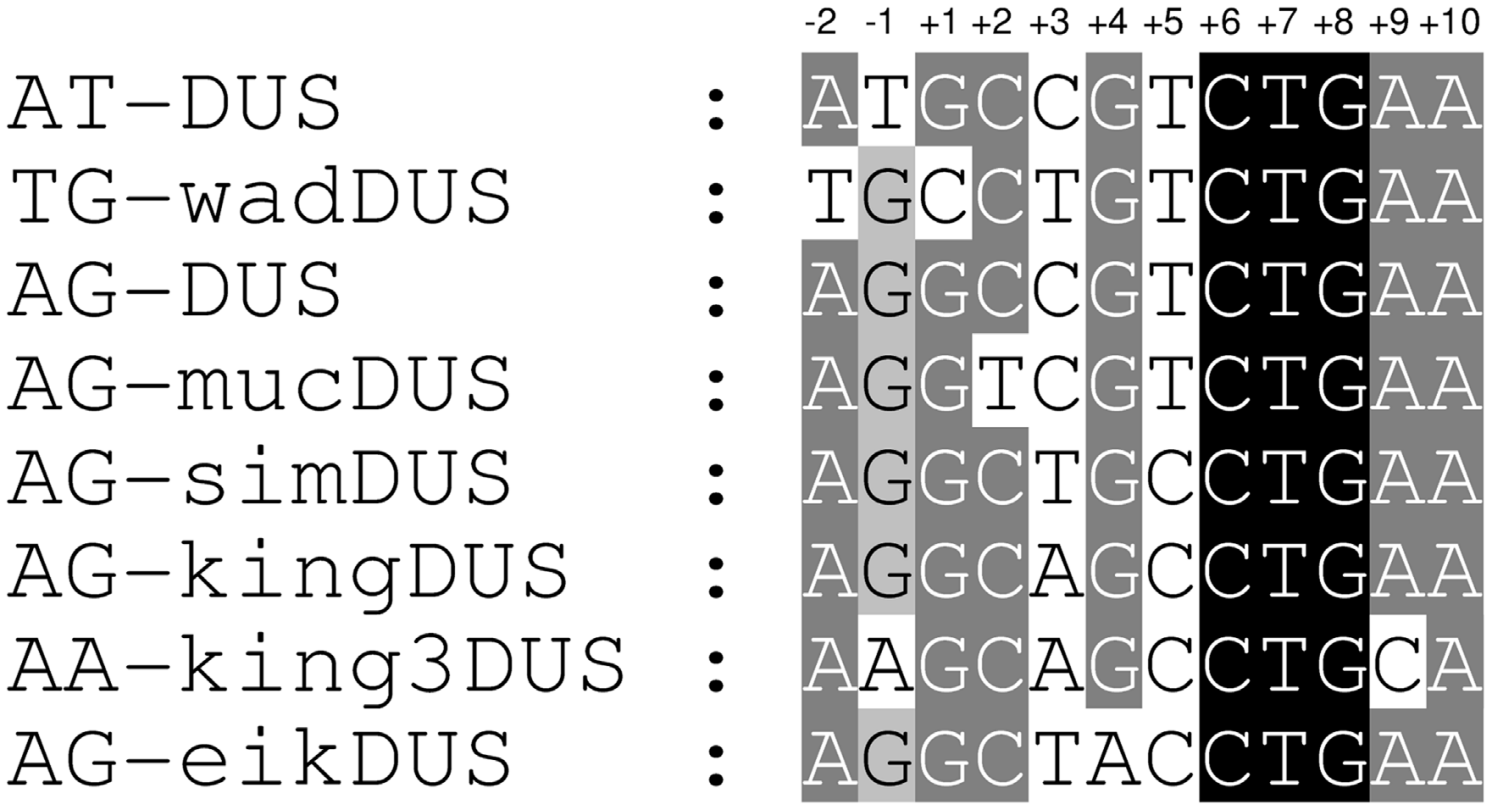
Alignment of eight distinct DUS dialects. Alignment of the eight different DUS dialects identified in the genomes of *Neisseriaceae* family members. Two nucleotides at the 5′-end of DUS are included and the numbering is given on the top. Relative shading indicate the level of sequence conservation.

*Neisseriaceae* genomes were obtained from online databases (i.e., the Human Microbiome Project [26] and other initiatives) and searched for overrepresented/highly-repeated sequences. Several very overrepresented 10-mers were identified in different genomes that displayed high degrees of similarity to the canonical DUS sequence first described in *N. gonorrhoeae* [7], [12], [13]. Eight distinct and abundant variants of DUS were identified and are shown in Figure 1, five of which are potential DUS as they were not previously functionally confirmed. The DUS variants are called dialects in concordance with previous use of the term for describing variants of short DNA motifs that probably are strongly affected by some DNA template-dependent processing proteins [24], and each DUS dialect was given a name according to the nomenclature scheme described in materials and methods. Every DUS dialect was found in exceptionally high numbers in their respective genomes and the exact occurrences are presented in Table S1 together with the number of degenerate DUS in which one nucleotide position were permitted to vary. AT-DUS was found in all available *N. gonorrhoeae* and *N. meningitidis* genomes (10-mer: n≈1900). In addition, AT-DUS was found as the most overrepresented repeat in the genomes of *N. lactamica* (n≈2200), *N. cinerea* strain ATCC 14685 (n = 943) and *N. polysaccharea* strain ATCC 43768 (n = 2183). AG-DUS was identified in *Neisseria sp*. oral taxon 014 (n = 3236), *N. subflava* strain NJ9703 (n = 2871), *N. flavescens* (n = 1196 and n = 2767 in strains NRL30031 H210 and SK114, respectively), *N. mucosa* strain C102 (n = 2964), *N. bacilliformis* strain ATCC BAA-1200 (n = 4265), *N. weaveri* strains ATCC 51223 and LMG 5135 (n≈2850), and *N. elongata* subsp. *glycolytica* strain ATCC 29315 (n = 3273). AG-mucDUS was the most prevalent repeat in *N. mucosa* strain ATCC25996 (n = 1543), *N. sicca* (n = 3770) and *N. macacae* strain ATCC 33926 (n = 3729) (Table S1).

A previous study showed that in *N. subflava* strain ATCC 19243, a 7 kb long sequence harbouring *folP* (GeneBank AJ581792.1) contained 7 mucDUS and 1 AG-DUS [22], whereas the genome of the *N. subflava* NJ9703 strain investigated here contained mainly the AG-DUS. The *folP* fragment is absent in the equivalent position in *N. subflava* NJ9703 and elsewhere in the genome. A BLAST search of all available *Neisseriaceae* genomes with the 7 kb fragment showed that the mucDUS positions around *folP* in *N. subflava* ATCC 19243 were present in *N. sicca*.

The genome of *N. wadsworthii* 9715 displayed a distinct DUS dialect, wadDUS (n = 2426), which is identical to AT-DUS with a T insertion after position +3 (Figure S1A).

In the genome of *K. oralis* ATCC 51147, yet another new dialect was discovered, the kingDUS (n = 5918). The occurrence of nearly six thousand kingDUS in a single small genome (2.4 Mb) is the highest density of any DUS dialect detected so far. By allowing a single nucleotide divergence in the kingDUS, the number of kingDUS-similar sequences increased by 21% to a total 7153 hits for the *K. oralis* genome. The completion, closure and annotation of the *K. oralis* genome may eventually alter the absolute numbers of kingDUS present, but approximately 2,5% of this particular genome will still remain occupied by the kingDUS which is very high compared to the approximate 1% DUS occupancy in *N. meningitidis* and *N. gonorrhoeae* genomes.

The genome of *S. muelleri* ATCC 29453 displayed the simultaneous presence of two DUS dialects. The kingDUS (n = 2257) described above and a new dialect simDUS (n = 2292) were in the *S. muelleri* genome detected in nearly equal numbers with a total count of 4549. SimDUS differed from the kingDUS by an A/T transversion at position +3 (Figure 1).

The genome sequences of *K. kingae* ATCC 23330 and *K. denitrificans* ATCC 33394 also revealed a new dialect, king3DUS, which differed from the kingDUS in an A/C transversion in position +9 (Figure 1) and a G/A transition in position −1 (Figure 1). The king3DUS was present in 2787 and 3603 copies in the genomes of *K. kingae* ATCC 23330 and *K. denitrificans* ATCC 33394, respectively.

Finally, the most divergent dialect of DUS relative to the AT-DUS was identified in the genomes of *E. corrodens* ATCC 23834 (n = 3269) and *Neisseria shayeganii* 871 (n = 2245), termed eikDUS. Notably, eikDUS was the only DUS with an A in position +4 (Figure 1). All the different dialects of DUS were conserved in positions +6, +7, +8 (CTG) as well as +10 (A) as demonstrated in Figure 1.

Based on the available genome sequences of genus *Neisseria*, no genome was devoid of any dialect of DUS. In the family *Neisseriaceae*, however, five genomes were found not to contain an abundant repeat that was an obvious DUS; these were the genomes of *Laribacter hongkongensis* HLHK9, *Lutiella nitroferrum* 2002, *Pseudogulbenkiania sp.* NH8B, *Chromobacterium sp.* C-61 and *Chromobacterium violaceum* ATCC12472. Their respective over-represented 10-mers are listed in Table S1D. We noticed, however, that the DUS core sequence 5′-CTG-3′ was found as the reverse complement sequence 5′-CAG-3′ in the most over-represented 10-mer sequences from *Laribacter hongkongensis* and *C. violaceum* (Table S1D).

### *Neisseriaceae* phylogeny

**Figure 2:**
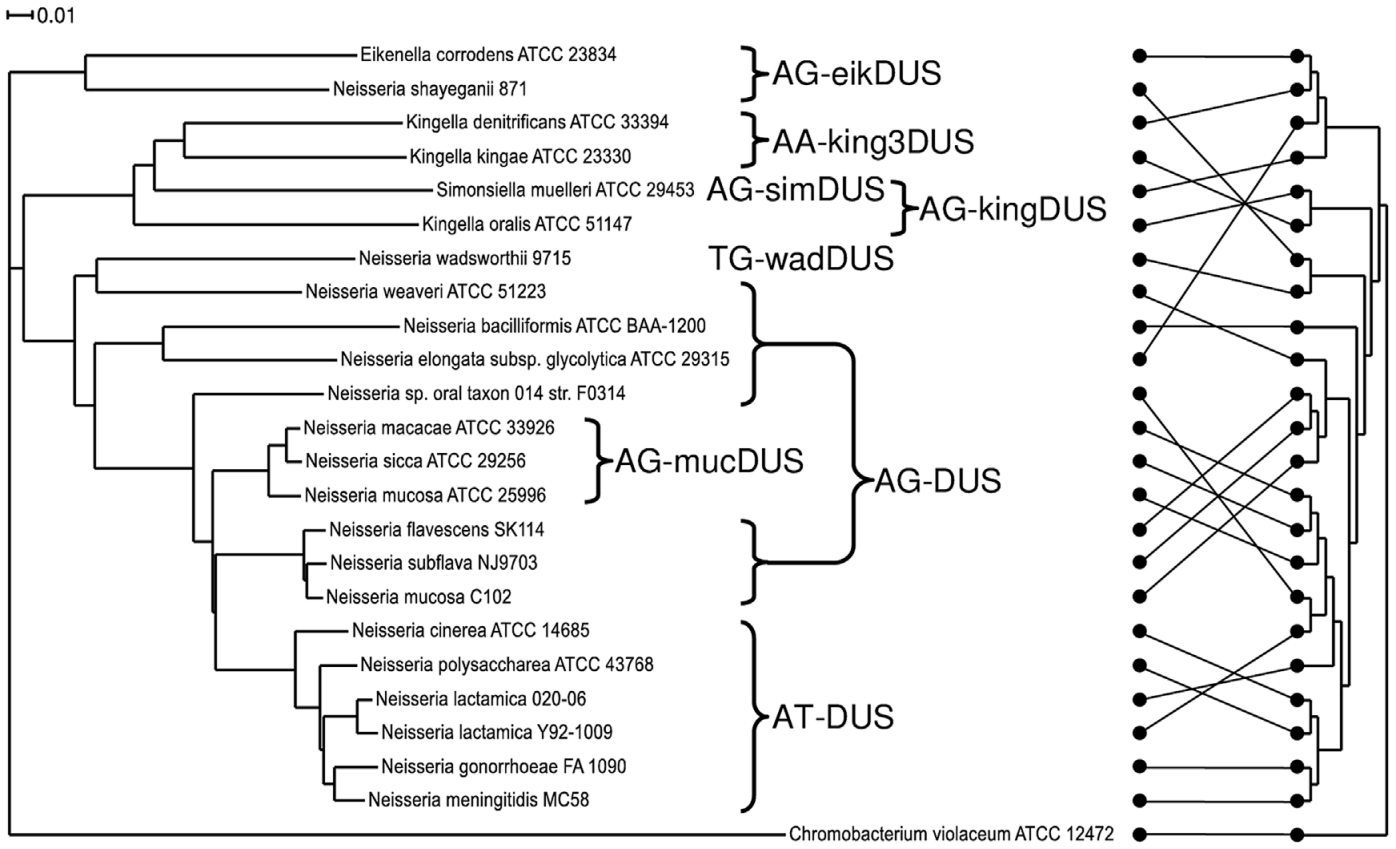
Phylogenetic tree based on the core genome and DUS dialect distribution. The phylogenetic tree obtained by the EDGAR service based on the core genome sequences of representative bacteria used in this study. The occurrences of the DUS dialects evolved are indicated. The scale bar represents 0.01 substitutions per nucleotide site. *Chromobacterium violaceum* was used as an outgroup. The core genome for this tree consisted of 474 coding sequences. On the right is the 16SrDNA based cladogram with the connectors of the dots showing the relation to the phylogram on the left.

Until now, a core genome phylogenetic tree for members of the *Neisseriaceae* was made only for the human genus *Neisseria* species [27], [28], [29], for all the available *N. meningitidis* genomes [25] and for collections of *Neisseria* strains [30], [31]. Here, a phylogenetic tree encompassing 23 representative members of the family *Neisseriaceae* was generated based on their common core genome containing 474 coding sequences (Figure 2). The *16SrDNA* phylogenetic tree [32], [33] made for the *Neisseriaceae* differed from the core genome based tree (Figure 2). Notably, the DUS dialect distribution in the two trees differed considerably, and the core genome tree branches reflected the presence of different dialects in a congruent manner. Also a phylogenetic tree based on ComP, a recently reported DUS-specific binding protein [18], displays high degree of congruence with different DUS dialects, although some deviations are apparent (Figure S7). The robust phylogeny finds that *N. shayeganii* 871 is closely related to *E. corrodens* ATCC 23834, and *S. muelleri* ATCC 29453 is located among the three different *Kingella* species. *Neisseria* sp. oral taxon 014 is in the *16SrDNA* tree wrongly placed close to the cluster containing *N. lactamica*. *N. mucosa* C102 is more closely related to *N. subflava* and *N. flavescens* than to *N. mucosa* ATCC 25996. The latter strain is the one in which the mucDUS was first described [28] and is located on the same branch in the *16SrDNA* phylogenetic tree as the type strain *N. mucosa* ATCC 19696, based on the available partial sequence (data not shown). This observation separated *N. mucosa* C102 from the *N. mucosa* ATCC 25996 reference strain. *C. violaceum* served as the outgroup in Figure 2, based on its suitable genomic distance from the other *Neisseriaceae* members.

The evolutionary history of DUS in *Neisseriaceae* may be traced and depicted as follows: The DUS-based transformation system evolved after the split from the shared common ancestor with *C. violaceum*. Neither could a ComP be indentified by BLAST searches of the genome of *C. violaceum.* Among the DUS-containing bacteria, the eikDUS-group separated first from the main branch, and is also the only group with an A in position +4 of the DUS. *N. shayeganii* strain 871 clusters with *E. corrodens* and may erroneously have been taxonomically assigned to the genus *Neisseria*. Thereafter, the kingDUS- and king3DUS-groups branched off from the canonical DUS-group and *S. muelleri* might have been in the process of separating itself from the *Kingella* group. *N. wadsworthii* separated from the AT-DUS-group by a change in DUS specificity evident from the insertion of a T in position +3 of AT-DUS. *N. macacae*, *N. sicca* and *N. mucosa* ATCC 25996 were separated from the AT-DUS- and AG-DUS-groups by the C/T transition in position +2.

### Single nucleotide transversion mutation analysis of AT-DUS in *N. meningitidis*

**Figure 3:**
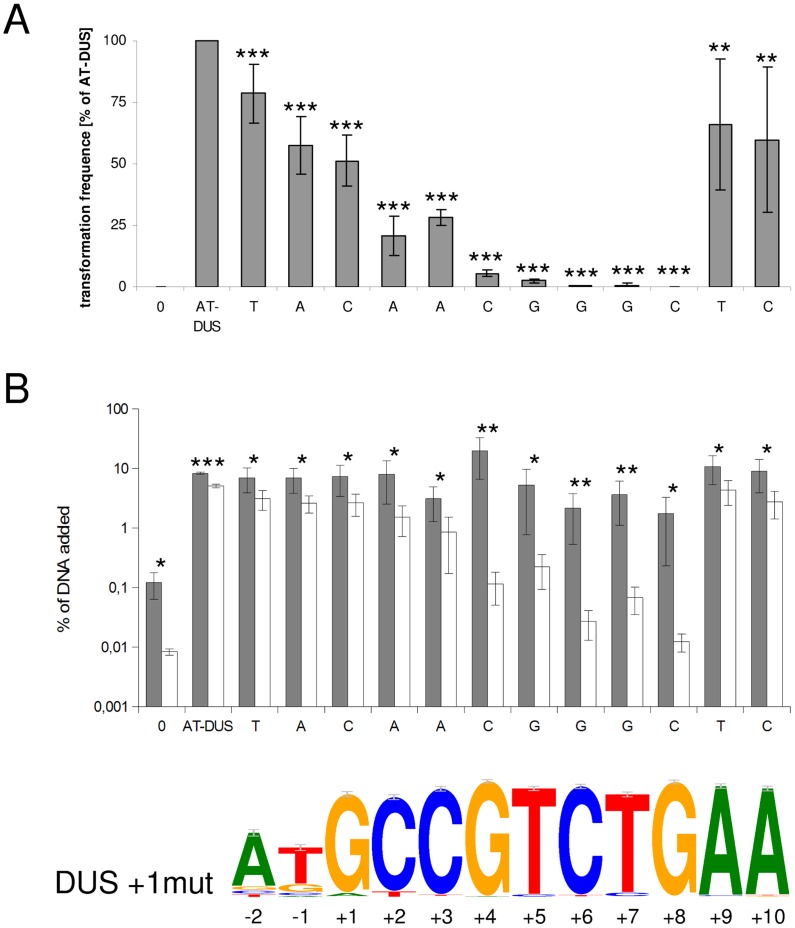
Effects of point mutations in the DUS on quantitative transformation and DNA binding and uptake of *N. meningitidis* MC58. (A) Quantitative transformation of *Neisseria meningitidis* strain MC58 with plasmid DNA containing a panel of modified DUS sequences. Transformation rates are shown as percentage relative to the AT-DUS. Standard deviations from 7 independent experiments are indicated by bars. (B) DNA binding (gray bars) and uptake (white bars) assay with radiolabelled plasmid DNA. Average values from 4 independent experiments are shown as percentage of total DNA added. T-test results for (A) transformation rates compared to AT-DUS and (B) for DNA binding versus uptake are indicated by stars (p≤0.2 = *, p≤0.05 = **, p≤0.001 = ***). DUS sequence transversions are given as abscissa labels. The sequence logo is based on the 2742 occurrences of DUS, with a single nucleotide divergence allowed, found in the genome of *N. meningitidis* MC58.

The new DUS dialects identified here exhibit several divergent positions (Figure 1) and we became interested in studying the discrete impact of the nucleotides that constitute a functional DUS. In a transversion mutation approach, the contribution of each individual nucleotide of the well-characterized AT-DUS was tested in quantitative transformation of *N. meningitidis* strain MC58 and the results are shown in Figure 3A. Also the effects of single transversion mutations in all twelve AT-DUS positions on DNA binding and uptake were measured and the results are summarized in Figure 3B. Any alteration of AT-DUS significantly reduced the transformation frequency (paired t-test, seven experiments, p≤0.02), although to a variable extent. The negative control lacking DUS does not transform at all. Our previous finding [14] was confirmed in that the two semi-conserved nucleotides in positions −2 and −1 at the 5′ end of the DUS, constituting the revised 12-mer AT-DUS, positively contribute to transformation efficacy, since both their respective transversions performed less than the complete AT-DUS. Furthermore, the transversions in individual positions of the 10-mer DUS were found to impair transformation performance. When the G in position +1 was transversed, the performance in transformation was reduced to 50% relative to the performance of the complete signal. Alterations in position +2 and +3 reduced the relative performance down to 20% and 28%, respectively. Alterations of position +4 (5%) and +5 (2%) had a more than one log reduction in relative transformation performance. The C, T and G at the 3′ half of the DUS (positions +6, +7 and +8) was shown to be particularly important for the DUS effect, since transformation was nearly abolished when the nucleotides in these positions were altered. A G/C transversion in position +8 gave rise to a total of only 2 CFU in seven experiments emphasizing the near complete loss of DUS-function. This functionally important 5′-CTG-3′ core is conserved in all dialects of DUS (Figure 1). The two adenines at the 3′ end of DUS (position +9 and +10) display minor contributions to the overall effect of DUS, and mutants perform at around 50% of the full AT-DUS. The A in position +10 is also conserved in all DUS dialects but contributes less significantly to the functionality of DUS than the 5′-CTG-3′ core. The distance-dependent gradual influence of the bases around the short 5′-CTG-3′ core sequence may reflect the strength of molecular interactions between DUS and the electropositive stripe on the surface of ComP [18] that warrants further investigations.

The effect of single transversion mutations on DNA binding and uptake in Figure 3B shows that DNA binding is high in *N. meningitidis* strain MC58 and that only DNA uptake is significantly affected. All the individual alterations of DUS bind better than the negative control lacking DUS. Relative DNA uptake was greatly reduced for the DNA without DUS and DUS with mutations in positions +4 to +8, being approximately 2% of bound DNA for the mutations in the core positions +6 to +8 (Figure 3B). In another strain, *N. meningitidis* 8013, DNA binding is lower overall and is together with the uptake negatively affected by the alterations of DUS. Again it is the alterations 5′-CTG-3′ that most dramatically affects DNA uptake and/or binding in both strains tested (Figure S4).

### DUS–mediated commutation in *Neisseriaceae*

**Figure 4:**
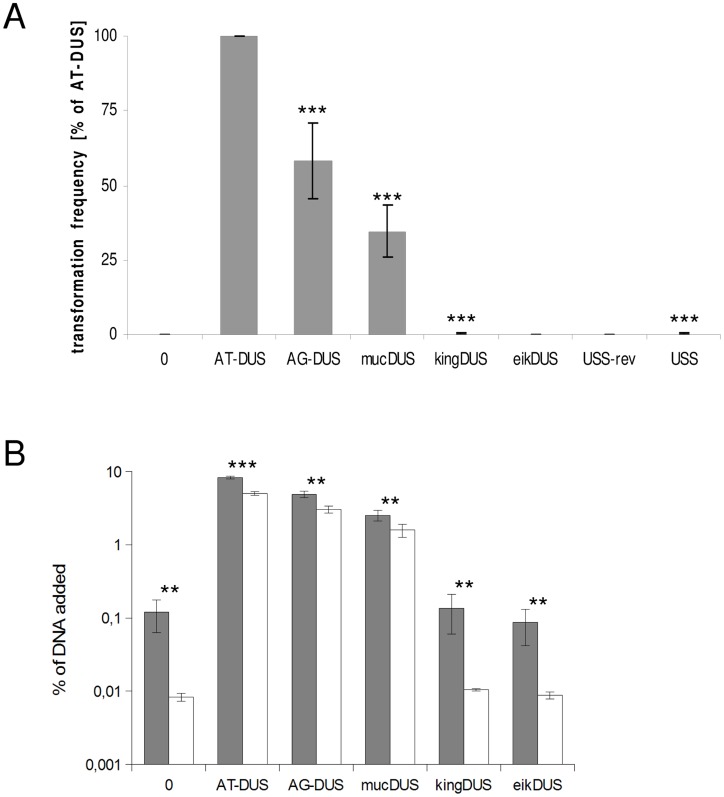
Quantitative transformation of *N. meningitidis* and binding and uptake of DNA with DUS from other *Neisseriaceae.* (A) Quantitative transformation of *Neisseria meningitidis* strain MC58 with plasmid DNA containing different DUS dialects and with the *Haemophilus influenzae* USS. Transformation rates are shown as percent relative to the AT-DUS. The standard deviations from 6 independent experiments are indicated by bars. (B) DNA binding (gray bars) and uptake (white bars) assay with radiolabelled plasmid DNA. Average values from 3 independent experiments are shown as percentage of total DNA added. Abscissa labels give the DUS variant. Statistic analysis as in Figure 3.

Potential commutation, defined as the interchange of DUS-linked genetic information, between different *Neisseriaceae* was first investigated by employing different DUS dialects in quantitative transformation experiments of *N. meningitidis* strain MC58, and the results are shown in Figure 4A and Table 1. Inversely, *K. denitrificans*, *E. corrodens* and *N. elongata* were tested for their respective DUS dependency by using PCR products of the *rpsL* gene conferring streptomycin resistance flanked by their own DUS or other DUS dialects. As shown in Table 1, the transformation frequency was always highest for their autologous DUS variant. The AG-DUS differs from AT-DUS in just a single nucleotide in position -1, and has 90% efficacy in *N. meningitidis* MC58. AG-mucDUS differs from AT-DUS in a T/G transversion in position −1 and a C/T transition in position +2. A 50% difference in transforming abilities of DUS and the 10-mer mucDUS in *N. meningitidis* was previously shown, although without statistical significance [22]. The 12-mer AG-mucDUS, which occurs 165 times in the genome of *N. meningitidis* strain MC58, displayed here a 66% reduced transformation efficacy relative to that of AT-DUS (Figure 4A and Table 1). For the more drastic C/A transversion in the second position (+2) in AT-DUS, a DUS-like sequence that occurred only once in the entire MC58 genome, the relative transformation was reduced by about 80% (Figure 3A). The AT-mucDUS was found 19 times, but the AG-mucDUS was found 88 times in the MC58 genome indicating previous interspecies transfer from the AG-mucDUS group. These transformation assays in *N. meningitidis* MC58 showed inter assay variations (Figure S3) but the Kendall's W test showed a very high concordance of gained orders (Kendall's W = 0.9145, χ2 = 44.8112, df = 7, p<0.0001).

**Table 1:** DUS–dependent transformation frequencies.

Strain	DNA	DUS	Frequency	Ratio^1^
*Neisseria meningitidis* MC58	pOHA-D4	AT-DUS	4,4E-06 (SD = 4,1E-06)	1
	pSAF-DV-48	AG-DUS	3,9E-06 (SD = 4,3E-06)	0,90
	pSAF-DV-49	AG-mucDUS	1,6E-06 (SD = 1,3E-06)	0,36
	pSAF-DV-50	AG-kingDUS	1,0E-09 (SD = 1,8E-09) 9 CFU in total^3^	0,0002
	pSAF-DV-51	AG-eikDUS	0^2^	n.a.^4^
	pSAF-DV-52	reverse USS	0^2^	n.a.^4^
	pSAF-DV-53	USS	2 CFU in total^3^	n.a.^4^
*Neisseria elongata* subsp. *glycolytica* 6171/75	*rpsL* - strep^R^	AG-DUS	3,1E-07 (SD = 2,4E-07)	1,39
	*rpsL* - strep^R^	AT-DUS	2,2E-07 (SD = 1,2E-07)	1
	*rpsL* - strep^R^	AG-mucDUS	9,2E-09 (SD = 1,5E-08)	0,04
*Kingella denitrificans* ATCC 33394	*rpsL* - strep^R^	AA-king3DUS	9,2E-06 (SD = 6,7E-06)	90
	*rpsL* - strep^R^	AT-DUS	1,0E-07 (SD = 6,8E-08)	1
*Eikenella corrodens* 31745	*rpsL* - strep^R^	AG-eikDUS	6,4E-05 (SD = 3,5E-05)	n.a.^4^
	*rpsL* - strep^R^	AT-DUS	0^2^	n.a.^4^

Frequencies of transformation in selected bacterial species with different DUS dialects displaying cross-species DUS transformation ratios. Experiments were performed at least three times.

1 transformation frequency relative to the AT-DUS transformation frequency in the same species.

2 erythromycin-resistant transformants could not be detected in any of all experiments.

3 cumulative number of CFU in all performed experiments.

4 not applicable.

SD = standard deviation.

AT-DUS containing DNA was completely unable to transform *E. corrodens*, the most phylogenetic distant DUS-containing species with the most divergent DUS dialect of the *Neisseriaceae*. *E. corrodens* was however readily transformed with its autogenic eikDUS documenting DUS-favoured transformation in the *Eikenella* genus for the first time. Also *K. denitrificans* transformed very poorly with AT-DUS and showed significant transformation with the autogenic king3DUS demonstrating DUS-favoured transformation in the *Kingella* genus for the first time. Low but significant transformation was achieved with AT-DUS in *N. elongata* harbouring the very similar AG-DUS. No biological transformation data was generated for *Neisseria mucosa* and *N. sicca* harbouring AG-mucDUS since the two strains tested, *Neisseria mucosa* type strain ATCC 19696 and *N. sicca* ATCC 29259, were not transformable with PCR-generated DNA or isogenic genomic DNA, both conferring streptomycin resistance.

**Figure 5:**
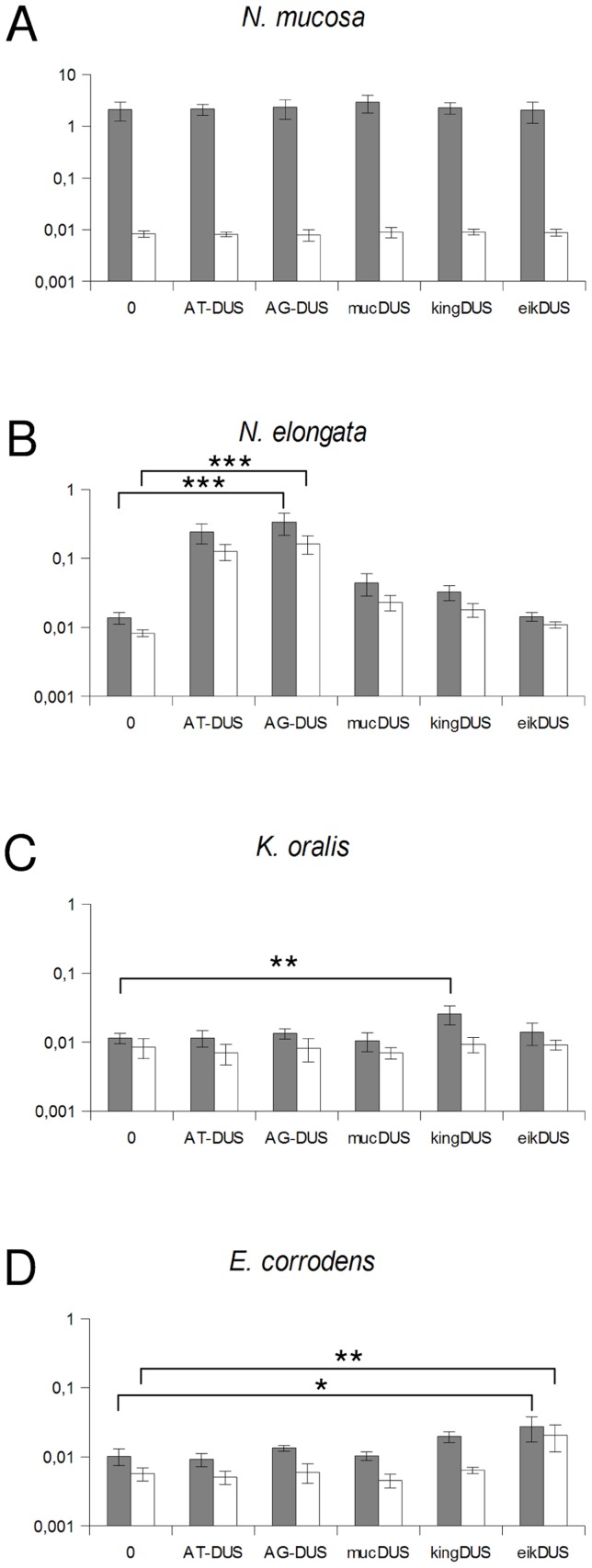
Quantification of DNA binding and uptake of *Neisseriaceae* with different DUS variants. Total DNA binding shown as gray bars and benzonase resistant DNA (uptake) shown as white bars plotted as percentage of DNA added. The species tested are *Neisseria mucosa* (A), *Neisseria elongata* subsp. *glycolytica* (B), *Kingella oralis* (C) and *Eikenella corrodens* (C). Abscissa labels give the DUS variant. Represented are the results from 3 (A, D) and 5 (B, C) independent experiments. Student's t-test values are indicated (p≤0.1 = *, p≤0.05 = **, p≤0.001 = ***).

Although the transformation efficiency is a measure of the final biological outcome, it is not useful for a quantitative measure of DNA binding to the cell and the DNA uptake. To assess the latter parameters in regard to the DUS dialects, the levels of binding and uptake of radiolabelled DNA was measured in different strains and species. In *N. meningitidis* MC58, binding of DNA with different DUS dialects was only reduced 1.7-fold and 3.3-fold for AG-DUS and AG-mucDUS, respectively, but about 60-fold for AG-kingDUS and about 95-fold for AG-eikDUS compared to AT-DUS (Figure 4B). DNA uptake was around 60% of bound DNA for AT-DUS, AG-DUS and AG-mucDUS but only around 10% of bound DNA for AG-kingDUS and AG-eikDUS. Another *N. meningitidis* strain, serogroup C strain 8013, was also tested and showed reduced overall sequence specific and dialect dependent DNA binding but did not show sequence specific DNA uptake (Figure S4 and Figure S5). ComP in these two meningococcal strains are identical, indicating that more factors influence DUS-dependent DNA uptake in these strains. *N. mucosa* ATCC 25996, *N. elongata* subsp. *glycolytica* ATCC 29315, *K. oralis* ATCC 51147 and *E. corrodens* ATCC 23834 were also tested for DNA binding and uptake (Figure 5). *N. mucosa* showed a DNA binding of >2% of added DNA while all other species tested displayed values <0.3%. Only *N. elongata* showed a DUS dialect-dependent DNA binding with 0.3% for its own AG-DUS and 0.01% for AG-eikDUS (Figure 5B). The binding and uptake performance of individual DUS-dialects in *N. elongata* mirrors those of *N. meningitidis* strain 8013 (Figure S5). The counts for *K. oralis* and *E. corrodens* were below 100 cpm and differences in performance of the different DNA templates were accordingly small. However, it is noteworthy that DNA binding of the autogenous DUS is significantly (p≤0.05) higher than the negative control in *K. oralis*. Similarly, the DNA uptake of the autogenous DUS is significantly (p≤0.05) higher than the negative control in *E. corrodens*. DNA uptake was around 60% of bound DNA for *N. elongata*, *K. oralis* and *E. corrodens* but only around 0.4% for *N. mucosa*. The results suggest that the investigated strains of these four bacterial species may not, or only to a small extent, carry out DUS-specific uptake of DNA (Figure 5) contrasting the clear transformation data (Table 1).

## Discussion

Genetic transformation in bacteria is a most common event in nature that requires a complex DNA uptake machinery and well-conserved recombination proteins [2], [34], [35], [36], [37]. Transformation distinguishes itself from other modes of horizontal gene transfer (conjugation and transduction) in that the recipient cell is actively taking part in the mobilization and integration of incoming DNA. Transformation in phylogenetically distant bacteria is thus adapted to ensure efficient uptake and recombination of homologous DNA [4]. This process relies on homologous recombination proteins whose processing functions are ubiquitously conserved from single celled bacteria to complex organisms, including humans. As such, transformation may be considered a low-complexity form of sex that is not firmly linked to reproduction but may have evolved to provide a similar selective advantage in breaking up associations among alleles [38]. The bacterial families *Neisseriaceae* and *Pasteurellaceae* are most suitable model organisms for the study of transformation and its role as a possible barrier to uptake of heterologous DNA. Here, we report the presence of eight distinct DUS dialects in the *Neisseriaceae* and correlate their distribution to the robust phylogeny of the family. This association emphasizes the influence of autogenic recombination on evolution and divergence of lineages. DUS-dependent transformation is documented for the first time in the genera *Eikenella* and *Kingella*. In a transversion mutant analysis the differential importance of each individual nucleotide that constitutes the AT-DUS was shown in *N. meningitidis* and these observations were found to relate to a conserved DUS core and the potential for interspecific DUS-mediated transformation.

### DUS counts

There are highly overrepresented sequences in the genomes of bacteria in general and the skewed occurrence of di-, tri- and tetra-mers has been particularly well documented [39]. These repeat distributions have proven valuable for classification [40]. The crossover hotspot instigator (Chi) sequence differs between bacterial species and new Chi sequences have been identified by a bioinformatics search for motifs [41]. Although uptake sequences and Chi sequences both are closely linked to homologous recombination, Chi and USS are distinctly different sequences in *H. influenzae* with no functional overlap [42]. As previously demonstrated [40], the DUS sequence is the most abundant repeat in the genomes of *N. meningitidis* and *N. gonorrhoeae* with about 1900 occurrences in the 2.2–2.3 Mb genomes (Table S1). In the *Neisseria sp.* containing the AG-DUS dialect, the counts were generally higher, around 3000. The most frequent DUS dialect was found for the kingDUS in *K. oralis* ATCC 51147 with nearly 6000 kingDUS within its 2.4 Mb genome sequence. Despite the high numbers of accurate DUS hits, the numbers of DUS with a single nucleotide divergence were considerably higher in all species (Table S1), revealing a potential for the activation of even more DUS positions.

The difference in total DUS count and ratio of DUS to genome size may reflect an ultimate saturation-state of DUS or indicate that this state has not yet been reached. Also, if DUS specificity was for some reason lost, the DUS could be degenerating slowly but progressively, as observed for pseudogenes. Bacterial genomes with a high number of DUS had a relatively low number of DUS with a single divergence (DUS+1mut) and *vice versa*. For example, *Neisseria bacilliformis* ATCC BAA-1200 had 4265 DUS and 4914 DUS+1mut (ratio 1∶1.15) while *Neisseria cinerea* ATCC 14685 had 943 DUS and 1372 DUS+1mut (ratio 1∶1.45) (Table S1). These differences could reflect differences in DUS dependency, which is known to vary in different *N. gonorrhoeae* strains, and may therefore also vary between species and their respective dialects [15]. Future studies will seek to address the influence of sequence variation and regulation of ComP and its antagonist PilV [17] in this regard. DUS saturation of the chromosome may also be opposed by factors such as the degree of interference with coding ability for intragenic DUS. Notably, all DUS dialects, except king3DUS, harboured a stop codon (UGA) in one reading frame, which imposes an obvious limitation on the liberty of positioning DUS.

By exploiting two different DUS dialects simultaneously some flexibility may be achieved in regard to which amino acids that are encoded by intragenic DUS. The genome sequence of *S. muelleri* harboured both the kingDUS and the simDUS, allowing for the variation of Q↔L and S↔C at the protein level when a DUS is found within a coding sequence. However, other explanations for the co-occurrence of two DUS-dialects in a single genome are high frequency of commutation between species (simDUS and kingDUS differ in a single nucleotide only), or consecutive habitats in different mammalian hosts with access to variant DUS dialects. DUS specificity may be altered by mutations in *comP* or by the acquisition of alleles encoding a novel DUS dialect, or simply by the presence of two DUS-specific proteins with different affinities, which could have originated from a simple gene duplication. However, only a single copy of *comP* was identified in the *S. muelleri* genome. It is also noteworthy that 1294 occurrences of the simDUS and kingDUS in *S. muelleri* are arranged as overlapping pairs in a dyad symmetry structure (Figure S6), which may indicate a dimer-based mechanism of DUS recognition. No preference for a single reading frame or positioning inside or outside of coding sequences was obvious when using the preliminarily annotated genome sequence from *S. muelleri*. A similar symmetry is found also in *Kingella* species where king3DUS pairs with king2DUS. The latter showed positive influence on transformation (data not shown) but is not a commonly found DUS by itself (Figure S6).

DUS have been found to locate in permissive regions of the core genomes of *N. meningitidis*, *N. gonorrhoeae* and *N. lactamica*, and intragenic DUS positions are common allowing them to be transcribed [11]. Intergenic regions, on the other hand, are particularly permissive and DUS sequences have been found to associate with transcriptional terminators by having frequently adopted an inverted paired organization, able to form stem-loop structures on ssDNA [7], [14], [43]. This inverted pair organization was found in high numbers in all genomes harbouring DUS dialects, suggestive of their association to transcriptional terminators (Table S2). In contrast to the simDUS and kingDUS arrangement in the *S. muelleri* genome, the individual DUS in an inverted pair DUS do not overlap.

Another interesting observation is the occurrence of peregrine DUS, exemplified by the mucDUS in Table S1A. *N. meningitidis* and *N. gonorrhoeae* genomes had a very consistent mucDUS count of about 160 and 110, respectively, while *Neisseria* sp. oral taxon 014 st. F0314 had the highest mucDUS count (467), and the *N. weaveri* strains had the lowest counts (15) in the canonical DUS group. The *N. meningitidis* genome for example contained 8% mucDUS in addition to the canonical DUS, while the genomes of the mucDUS-containing group of bacteria harboured between 7% and 12% DUS. These numbers likely reflect recent commutation between these two groups sharing the same ecological niche. The exchange of highly selectable markers between *N. meningitidis* and the commensal *Neisseriae* is well established [44], [45], [46], [47], [48], [49]. The amount of mucDUS relative to the canonical DUS was particularly low in the *N. weaveri* genomes (0.5%, Table S1A), which is interesting since *N. weaveri* is a canine commensal and only an opportunistic pathogen to humans [31], [50], [51].

In contrast to a previous report on the abundance of mucDUS in *N. subflava* strain ATCC 19243, we found that AG-DUS is the most abundant dialect in *N. subflava* NJ9703 [22]. Possibly, *N. subflava* strain ATCC 19243 acquired the *folP* sequence fragment from *N. sicca* and therefore harbours the mucDUS in this region, or alternatively, *N. subflava* strain ATCC 19243 is more closely related to *N. sicca* than to *N. subflava* NJ9703. We identified more canonical DUS in the *N. elongata* subsp. *glycolytica* strain ATCC 29315, 3273 as opposed to 2142 than in the former study by Higashi *et al.* [23], and more mucDUS, 174 as opposed to 117. These differences could possibly be due to recent updates of the genome sequence files available. Comparison of the predominantly mucDUS-containing bacteria is difficult, as Higashi *et al.* did not specify which strains were analysed, both were, however, reported to contain >3400 copies of the mucDUS. In contrast to this observation, we identified only 1543 mucDUS in *N. mucosa* strain ATCC 25996 while *N. mucosa* C102 had only 155 mucDUS and 2964 canonical DUS. These discrepancies indicate that strain C102 may erroneously be assigned *N. mucosa*, also since both the core genome phylogeny (Figure 2) and that of ComP demonstrated the close genetic relationship between this strain and *N. subflava* and *N. flavescens*.

### Core genome-based phylogeny

*Neisseriaceae* are highly recombinogenic yielding a polyphyletic family structure, and resolving the family into distinct species was achieved by including large amounts of sequence data in the analysis. Initially, such analyses were based on sequence divergence of a single gene (*16SrDNA*) or on a small number of housekeeping genes as in multi locus sequence typing (MLST) [52].

The evolution of distinct DUS dialects in this phylogenetically compact family is a striking example of how preference for homologous DNA in highly transformable bacteria affects evolution. Differences between the dialects are expected to be mirrored in the amino acid sequence(s) of the recently confirmed DUS-specific binding protein ComP, and warrants further functional investigation. The congruence between DUS-dialect and phylogeny and the presence of ComP suggests that those dialects that remain to be confirmed functional DUS are true DUS. This is also further emphasized by the exceptional overrepresentation and conservation of each dialect in their respective genomes. The most plausible hypothesis explaining these observations is DUS-dependent bias in frequent transformation/recombination [11].

### Single nucleotide mutation analysis of DUS

The differential influence of each nucleotide in AT-DUS on *N. meningitidis* transformation and DNA binding/uptake was tested by employing donor DNA harbouring altered DUS. Single nucleotides were altered to be the transverse (purine↔pyrimidine) and the least common nucleotide at that position in the *N. meningitidis* genome. A similar analysis, based on the uptake of radioactive labelled DNA, has previously been reported in *H. influenzae* [53], [54]. Here the first steps of transformation were investigated by a DNA binding and uptake assay. The quantitative transformation method employed here measures the outcome of both uptake and recombination of DNA. This gradual analysis is important since it has been documented that DUS may influence multiple steps during transformation [15]. The most significant 5′-CTG-3′ core identified in transformation was conserved in all dialects of DUS (Figure 1). In contrast to the transformation experiments with *N. meningitidis* MC58, DNA binding did not display differential binding of AT-DUS and mutated AT-DUS versions, but clearly showed that binding discriminated against the DUS-less negative control (Figure 3B). This observation suggests that DNA binding in this strain is not very strict in terms of DUS specificity, and that DUS and single nucleotide mutated DUS can contribute to binding. Also the observation that the DUS dialects most similar to AT-DUS bound better than the more distant dialects emphasizes this point (Figure 4B).

It has been hypothesized that DUS specificity may function at more than one level during transformation [15] and one may speculate that initial binding by ComP [17], [18] could display weak DUS specificity and that the influence of the core 5′-CTG-3′ first become influential during uptake or later during the transformation process. The DNA uptake data from *N. meningitidis* (Figure 3B and Figure 4B) are corroborating the transformation data in that AT-DUS outperforms the other dialects tested. The differences in relative uptake of the close and distant DUS dialects in *N. meningitidis* (Figure 4B) suggest that DNA uptake is not only a relative function of binding, but may be influenced by DUS specificity.

### DUS–mediated commutation in *Neisseriaceae* species

The transformation performance of individual DUS-dialects from separate phylogenetic branches (Figure 2) was tested in *N. meningitdis*, *N. elongata*, *E. corrodens* and *K. denitirificans* (Table 1). The degree of similarity between the DUS-dialect of the recipient species, AT-DUS in *N. meningitidis*, and that in the donor DNA, AG-DUS, AG-mucDUS, AG-kingDUS and AG-eikDUS, directly correlate with the level of transformation. The potential for high levels of commutation when DUS dialects are similar is reflected in reports describing interchange of DNA between pathogenic and commensal *Neisseria in vivo* [45], [55].

This correlation is also evident in transformations of the AG-DUS species *N. elongata*, since AT-DUS outperforms AG-mucDUS. These observations suggest further that a nucleotide change in position −1 of the DUS is less influential than a change in position +2 in concordance with the results in *N. meningitidis* (Figure 3A). Transformations in the genera *Eikenella* and *Kingella* show strict autologous DUS-dependency in transformation indicating that AT-DUS is too divergent to allow transformation. It is well established that general sequence divergence between recipient chromosome and transforming DNA is strongly affecting homologous recombination, the last step in transformation [3]. Based on these observations one may anticipate that the phylogenetic distance correlates with the potential for commutation since DUS dialect distribution is reflected in the orthology of the *Neisseriaceae*. Furthermore, no significant transformation of *N. meningitidis* was observed when transforming DNA carried USS, which is the DUS of the *Pasteurellaceae*. Since *H. influenzae* and *N. meningitidis* share the same habitats, and are likely to encounter each other's DNA in e.g. oropharyngeal biofilms, the establishment of a functional barrier to commutation between these species may be important for the preservation of genome integrity. Genetic exchange between *N. meningitidis* and *H. influenzae* is rare [56] while the frequent commutation within the *Pasteurellaceae* is well documented [21], [57].

*N. elongata* subsp. *glycolytica* was previously shown to be transformable with a GT-mucDUS, but with an 8-fold reduced efficacy compared to a GT-DUS [23]. In our analysis, using a similar reporter construct, this factor was higher for the AG-mucDUS when compared to the AT-DUS (25-fold) and when compared to the AG-DUS (35-fold) (Table 1). These differences could relate to the employment of the non-ideal GT-DUS in the initial study. The DNA binding and uptake assays show that *N. elongata* subsp. *glycolytica*, like *N. meningitidis*, binds DNA in a DUS-specific manner with preference for the most similar DUS sequence corroborating the transformation results discussed above.

The influence of AG-DUS and AT-DUS in transformation of *N. mucosa* or *N. sicca* could not be tested since these strains were incompetent for transformation. The molecular basis for this remains unexplored, but the DNA uptake data show that transformation deficiency can be linked to the reduced ability to take up DNA, suggestive of a malfunction in this initial step of transformation. It is curious that *N. mucosa* binds DNA exceptionally well in a DUS-independent manner and this observation warrants further investigation. The functionality of AG-mucDUS in transformation has also been verified in other laboratories (N. Weyand, personal communication, [22]) but mucDUS-dependent transformation of species in the mucDUS-group has not yet been demonstrated. Recent observations in our lab confirm that also wadDUS is a true DUS affecting transformation in *N. wadsworthii* (unpublished data). The simDUS found in *S. muelleri* and kingDUS found in *K. oralis* are the only DUS that remains to be functionally verified, but the presence of *comP* genes in their respective genomes [18], the high overrepresentation of each DUS dialect and intragenomic DUS conservation in addition to their high similarity to the other DUS strongly suggests that they are, or at least have been, genuine DUS.

*E. corrodens* and *K. denitrificans* have previously been shown competent for transformation with homospecific DNA [58], [59]. Here, we established that this specificity is DUS-dependent. The DNA binding and uptake results did not reflect the differences observed in transformation since DNA binding was relatively uniform irrespective of the DUS-dialect used. However, it must be noted that binding was very low in both *K. oralis* and *E. corrodens* although a weak but statistically significant preference for autologous DUS in DNA binding or uptake, respectively, is evident (Figure 5).

Here, we expanded the number of bacteria that utilize a DUS-dependent mechanism for transformation of homologous DNA. Eight distinct dialects of DUS in the family *Neisseriaceae* were described, and the ability to overcome transformation barriers was assayed by both transformation and DNA binding and uptake for five of these. Furthermore, the evolution of DUS dialects corresponds with the evolution of the distinct core genomes of each phylogenetic clade. The DUS signal was analyzed by single nucleotide mutational analysis and an essential three nucleotide core sequence was found to be strictly required for transformation. This functional DUS core is conserved in all eight DUS dialects. The level of commutation was found to correlate with the phylogenetic distance and also to the similarity of the DUS sequences themselves. Future studies will explore the evolution of DUS dialects in regard of the recently confirmed association between DUS and ComP.

## Materials and Methods

### Genome sequence bioinformatic analysis

#### Search for overrepresented repeat sequences

Sequences from completed and unfinished *Neisseriaceae* genomes were retrieved from NCBI GenBank. For unfinished sequences, the available contigs were concatenated into a single FASTA file. The Perl script repeat_finder.pl [21] was used for the in silico search for overrepresented sequences in these files. Since DUS and USS reportedly are 12 and 9 base pairs long, respectively, this range was used for the search. Highly overrepresented sequences were aligned to find repeated patterns and similarities to known repeats. The program fuzznuc from the EMBOSS package [60] was used to count the number of occurrences of new identified repeated sequences, allowing also for nucleotide variations (see Table S1). An in-house Perl-script was used to extract the repeat sequences including surrounding sequence into a FASTA file, which was used as input for the WebLogo service [61], to generate the sequence logos shown in Figure S1. To clearly distinguish between new DUS dialects and existing terms, a nomenclature scheme was devised. The basic name DUS was applied to 10-mer DNA Uptake Sequences in general and specifically assigned to the canonical 10-mer DUS found in *N. meningitidis* and individual nucleotides were numbered 1–10 as in previous publications. Capital letters in front of DUS-name depict conserved bases in positions −1 and −2 such as AT in the 12-mer AT-DUS in concordance with previous use [14], [15]. The new DUS dialects were assigned an abbreviated name of the host species, in which the variant was first found, in small letters in front of DUS (Figure 1). The eikDUS was first identified in the genome of *Eikenella corrodens*, the kingDUS in *Kingella oralis*, the king3DUS in *Kingella denitrificans*, the simDUS in *Simonsiella muelleri* and the wadDUS in *N. wadsworthii*. The mucDUS, which was previously called “altered DUS” or “DUS1” [22], [23], was first identified in *N. mucosa*.

#### Construction of a core genome-based phylogenetic tree

A phylogenetic tree for the family *Neisseriaceae* was made using the annotated completed genomes and annotated contigs from Genbank. In addition the sequences from *Neisseria* sp., oral taxon 014 and *S. muelleri* ATCC 29453 were annotated with GenDB [62]. *C. violaceum* ATCC 12472 was used as the outgroup in the phylogenetic analysis. With the availability of genome sequences it was now possible to establish a phylogeny based on whole genome alignments. These comprehensive alignments provide information about the pan-genome and the core genome and thereby allow the establishment of a very robust phylogeny. Many bioinformatics tools were designed that can be used for this task [63], [64], [65], [66]. With the increasing number of genome sequences, highly stable core genome phylogenetic trees were generated for different groups of the genus *Neisseria* [25], [27], [28], [29], [31]. Here, we used the EDGAR service to build the phylogenetic unrooted tree based on 474 coding sequences constituting the core genome of 22 bacteria [67].

### Construction and purification of plasmids containing the DUS variants

Plasmids containing the dialects and transversion variants of DUS were based on the plasmid p0-DUS [14]. Oligonucleotides listed in Table S3 were used to amplify the *pilG::ermC* fragment from p0-DUS by PCR whereby the oligomer OH3 was always used as reverse primer. The PCR products were digested with *Xho*I and *Sac*II and inserted into the multiple cloning site of the vector pBluescript II SK+ (Stratagene, USA). *E. coli* strain ER2566 (NEB, USA) was used for cloning and the strain XL-1 Blue (Stratagene, USA) was used for large scale purification of plasmids due to higher yields. Plasmids were purified using the QIAGEN Plasmid Plus Midi Kit (Qiagen, Germany). Plasmids are listed in Table S4. The DNA was diluted to a concentration of 100 ng/µl in 10 mM Tris, pH 8, and stored at −20°C until used.

### Bacterial strains and growth conditions

Bacteria used in this study are listed in Table S4. *Escherichia coli* strains were grown on LB medium. *E. coli* XL-1 blue was used for the quantitative production of the pDV plasmids. *N. meningitidis* and *N. elongata* were grown on blood agar plates, on GC medium plates or in liquid GC medium supplemented with IsoVitaleX. *K. denitrificans* and *E. corrodens* were grown on blood or chocolate agar plates or, when in liquid, in brain heart infusion broth. Antibiotics were added to the media when appropriate.

### Quantitative transformation

Quantitative transformation was performed as previously described [14] using plasmid or genomic DNA carrying an antibiotic resistance marker. Briefly, for *N. meningitidis*, cells grown over night at 37°C were suspended in 5% CO_2_ saturated GC medium containing IsoVitaleX and 7 mM MgCl_2_. 5 µl of DNA (100 ng/µl) were provided in 15 ml tubes, 500 µl cell suspension was added, shortly mixed by vortexing and incubated at 37°C for 30 min without agitation. Each sample was diluted by adding 4.5 ml GC medium and incubated for 4.5 h at 37°C on a tumbler (60 rpm). The cultures were then mixed and serial dilutions prepared in GC medium. Of each undiluted sample, 50 µl aliquots were spread on blood agar plates containing 8 µg ml^−1^ erythromycin or 50 µg ml^−1^ streptomycin and 50 µl of the 10^−7^ dilution were spread on blood agar plates without antibiotics. At least 2 agar plates were inoculated from each sample and experiments were repeated at least three times. Colonies were counted following over night incubation in a 5% CO_2_ atmosphere at 37°C. Individual transformation frequencies were calculated as the number of antibiotic-resistant colony forming units (CFU) per total CFU. The absolute transformation frequencies varied between experiments, possibly due to the difficulty in reproducing bacterial suspensions with identical fractions of competent bacteria. This problem was also reported earlier [22], and was resolved here by the use of relative values based on an internal standard. A Kendall's W test showed a very high concordance of gained orders (Kendall's W = 0.9346, χ2 = 85.0506, df = 13, p<0.0001). The absolute transformation frequencies are plotted in Figure S2.

In order to transform *N. elongata*, *K. denitrificans* and *E. corrodens*, small alterations of the protocol were required. The *N. elongata* subsp. *glycolytica* strain employed here was the type strain originally isolated by Henriksen *et al.* [68]. Agglutinating P+ colonies of *N. elongata* were pre-selected since previous experiments had documented a positive correlation between agglutination and transformation [69]. The type strain of *K. denitrificans* and *E. corrodens* strain 31745 were used for quantitative transformation. The *E. corrodens* strain 31745 is a reference strain that was previously shown to be transformable [58]. To generate donor DNA for the quantitative transformation experiments, spontaneous streptomycin-resistant mutants of *N. elongata*, *K. denitrificans* and *E. corrodens* were isolated and the genomic DNA from these strains used as a template for a PCR of the *rpsL* gene using primers listed in Table S5. The *rpsL* genes were sequenced, confirming the Lys to Arg mutation at amino acid position 43 conferring streptomycin resistance [70]. *rpsL* PCR products were adjusted to 50 ng/µl and 10 µl thereof was used in transformation of 500 µl cell suspension. *K. denitrificans* was transformed in Brain heart infusion medium supplemented with 7 mM MgCl_2_ and the second incubation time was extended to 5 hours.

### DNA binding and uptake assay with radiolabeled DNA substrates

For radiolabeling the pDV plasmids (Table S4) were linearized by digestion with *Sca*I, purified using QIAquick columns (Qiagen) and treated with exonuclease III (Fermentas). After heat inactivation 10 µg of the partially single stranded DNA were incubated with 3 µM dNTPs, 20 µCi [α-32P]dCTP and 10 units Klenow fragment (3′→5′ exo–) (NEB) for 90 min at 37°C. The fill-in reaction was finished after increasing the dNTP concentration to 30 µM and additional incubation for 30 min. The products were purified as before and showed specific activities of 4×10^5^ to 2×10^6^ CPM µg^−1^. The *Neisseriaceae* were grown in liquid medium to an optical density at 660 nm of approximately 1. MgCl_2_ was added to 7 mM and one ml aliquots were incubated with DNA with about 5×10^6^ CPM activity and rotated at 37°C for 45 min. The samples were then split into two 500 µl samples and 12.5 units Benzonase (Merck) was added to one of these. After additional incubation for 15 min the cells were washed three times by centrifugation for 3 min at 5000× g and resuspension in liquid medium including 7 mM MgCl_2_. The final pellets were resuspendet in 3 ml scintillation fluid (Ultima Gold MV, PerkinElmer) and measured twice for 3 min in a Tri-Carb 2900TR (PerkinElmer) using an energy window LL-UL = 50–1700. DNA binding and uptake are reported as the percentage of DNA added and percentage of cell-bound DNA, respectively, and the results are presented as the means of for 3 to 5 replicates.

## Supporting Information

Figure S1 Sequence logos of the DUS dialects. Sequence logos of DUS dialects identified in this study. The relevant 10-mer DUS was used for the search, allowing one divergence (except for *Simonsiella muelleri*, as it contains two different DUS). The four nucleotide positions on both sides are included. For the counts of sequences used see Table S1. A) AT-DUS and TG-wadDUS, B) AG-DUS, C) AG-DUS and AG-mucDUS, D) AG-eikDUS, AG-kingDUS and AA-king3DUS, and E) AG-kingDUS and simDUS.
(PDF)

Figure S2 Quantitative transformations of *N. meningitidis* MC58 with DUS containing point mutations. The graphs show the transformation frequencies used for Figure 3A. The variations between the seven experiments in the range are visible as well as the relative consistent relation to the internal standard AT-DUS.
(PDF)

Figure S3 Quantitative transformations of *N. meningitidis* MC58 with DUS variants. The graphs show the transformation frequencies from seven independent experiments which were used for Figure 4A. Range variations with consistent ranking are seen as in Figure S2.
(PDF)

Figure S4 Effects of point mutations in the DUS on DNA binding and uptake of *N. meningitidis* 8013. Equivalent to Figure 3B. Data derived with the *N. meningitidis* strain 8013. Quantification of the binding of radiolabelled DNA to live cells. Total DNA binding shown as gray bars and benzonase resistant DNA (uptake) shown as white bars plotted as percentage of DNA added. Average values from 4 independent experiments are shown and standard deviations are indicated by bars. Student's t-test results for DNA binding versus uptake are marked by stars (p≤0.2 = *, p≤0.05 = **, p≤0.001 = ***). DUS sequence transversions are given as abscissa labels. Sequence logo as in Figure 3.
(PDF)

Figure S5 Quantification of DNA binding and uptake of *N. meningitidis* 8013 with DUS from other *Neisseriaceae.* Equivalent to Figure 4B. Data derived with the *N. meningitidis* strain 8013. Total DNA binding shown as gray bars and benzonase resistant DNA (uptake) shown as white bars plotted as percentage of DNA added. Results form 3 independent experiments are represented. Abscissa labels give the DUS variant. Student's t-test values are indicated (p≤0.2 = *, p≤0.05 = **, p≤0.001 = ***).
(PDF)

Figure S6 Dyad symmetry structures. Graphical representation of the DUS containing dyad symmetry structures found in the genomes of *Simonsiella muelleri* ATCC 29453 (A) and *Kingella denitrificans* ATCC 33394 (B) and the number of their occurrences in selected genomes. The translations of the three coding frames are given on top with the variable positions shaded in gray.
(PDF)

Figure S7 Comparison of the core genome based and the ComP based phylogeny. The phylogenetic tree from Figure 2 (left) is compared to the ComP based tree (right) with connectors showing the relations. Homologues to *N. meningitidis* strain MC58 ComP were identified with EDGAR [67] and BLAST [71] and the ComP phylogram was based on a ClustalW generated alignment of the globular domain (residues 35–149) of ComP. The scale bars represent 0.01 substitutions per nucleotide site.
(PDF)

Table S1 Number of DUS sequences in *Neisseriaceae.* Counts of DUS in the genomes of species that contain A) the canonical DUS, B) the mucDUS, and C) other DUS variants, and D) other over-represented sequences in other *Neisseriaceae*. The overrepresented sequences were detected with the repeat-finder Perl script [21] and counts obtained using fuzznuc [60].
(PDF)

Table S2 Inverted DUS repeats in *Neisseriaceae.* Counts of DUS containing inverted repeats in the genomes of selected *Neisseriaceae* species.
(PDF)

Table S3 DUS variation oligonucleotides. Primers employed in the PCR based amplification of the *pilG*::*ermC* fragment from the plasmid p0-DUS.
(PDF)

Table S4 Plasmids and bacteria. Bacterial strains and plasmids employed in this study.
(PDF)

Table S5 *RpsL* specific PCR primer. Primers employed for the PCR-based amplification of the *rpsL* gene.
(PDF)
