# Supplementary material for: Dialects of the DNA Uptake Sequence in Neisseriaceae
Source: PLoS Genet. 2013 Apr 18;9(4):e1003458. doi: 10.1371/journal.pgen.1003458 (PMC3630211; doi:10.1371/journal.pgen.1003458)
Supplement: Figure S1 — Sequence logos of the DUS dialects. Sequence logos of DUS dialects identified in this study. The relevant 10-mer DUS was used for the search, allowing one divergence (except for Simonsiella muelleri, as it contains two different DUS). The four nucleotide positions on both sides are included. For the counts of sequences used see Table S1. A) AT-DUS and TG-wadDUS, B) AG-DUS, C) AG-DUS and AG-mucDUS, D) AG-eikDUS, AG-kingDUS and AA-king3DUS, and E) AG-kingDUS and simDUS. (PDF) [file pgen.1003458.s001.pdf]

A

Neisseria meningitidis MC58 (AT-DUS)

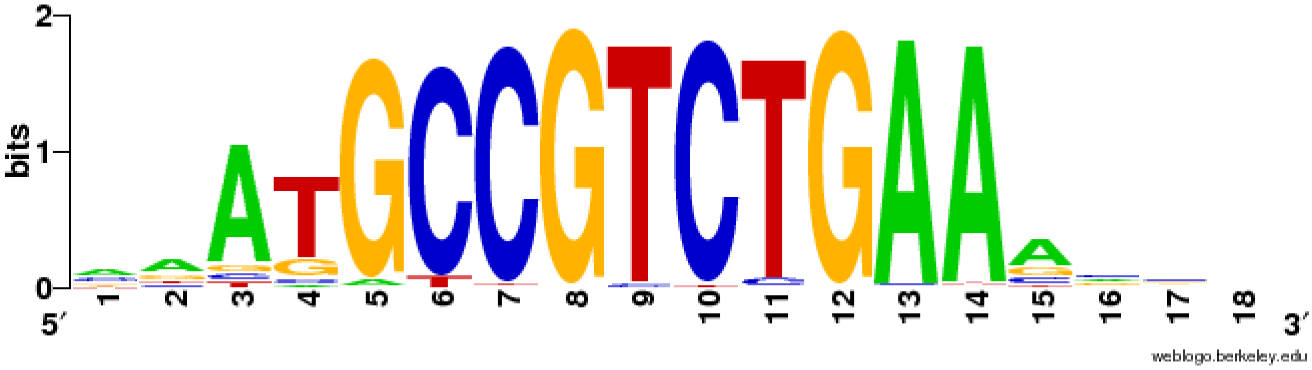

Neisseria lactamica Y92-1009 (AT-DUS)

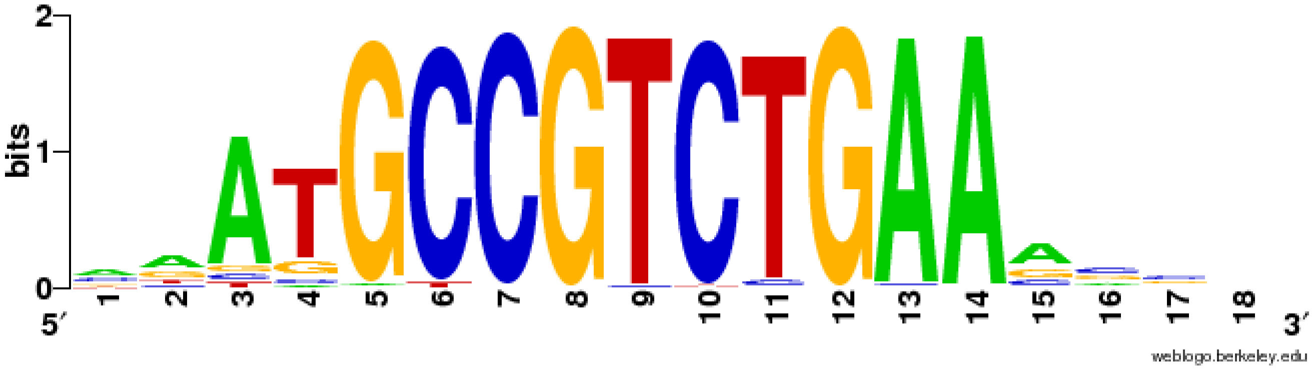

Neisseria wadsworthii 9715 (TG-wadDUS)

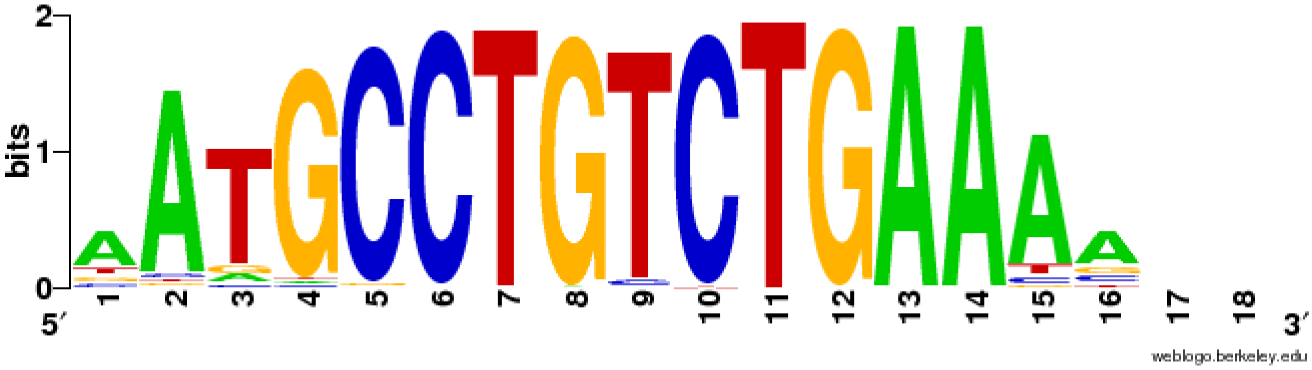

Figure S1: Sequence logos, single divergence permitted

B

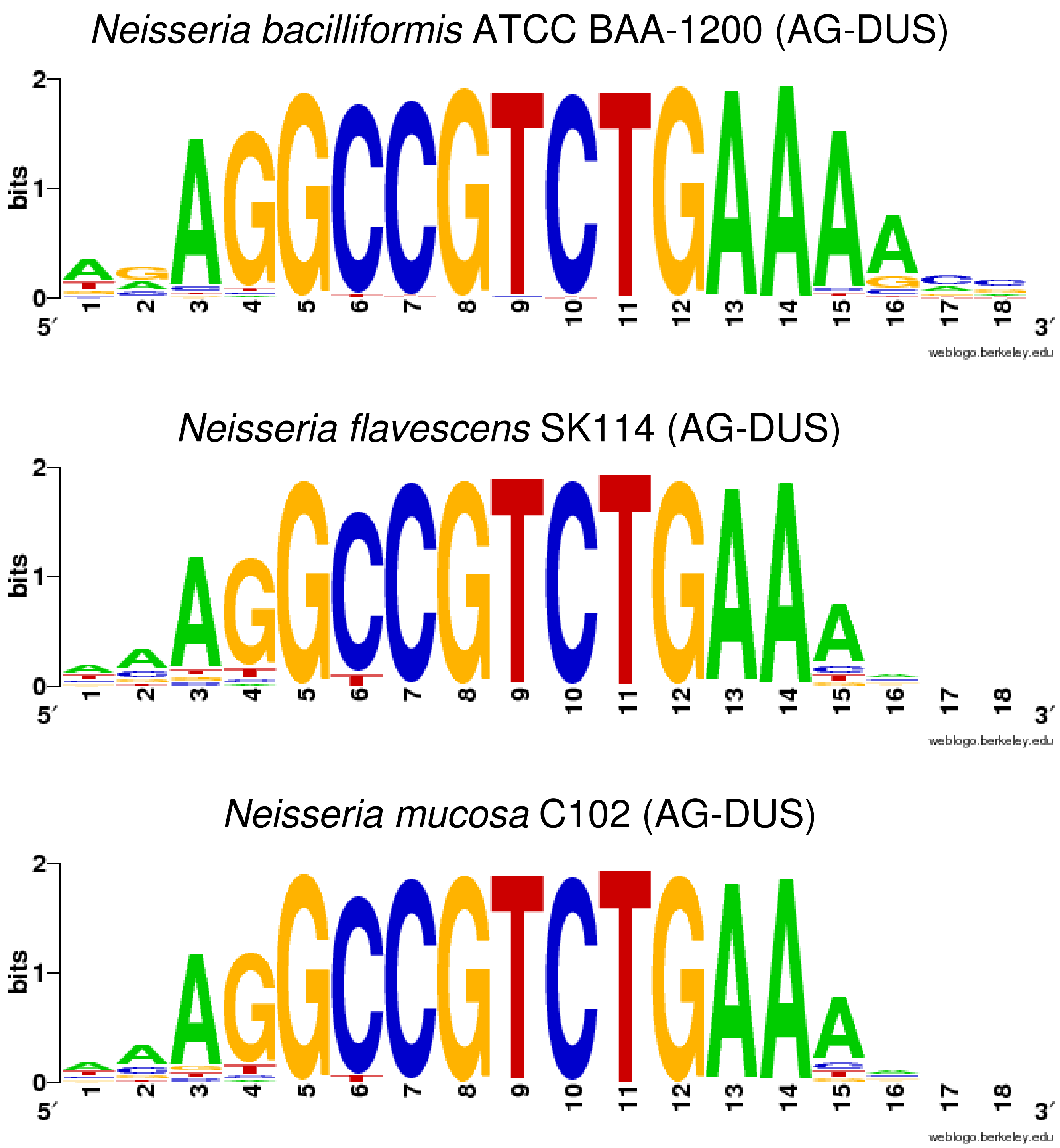

Figure S1: Sequence logos, single divergence permitted

C

Neisseria elongata subsp. *glycolytica* ATCC 29315 (AG-DUS)

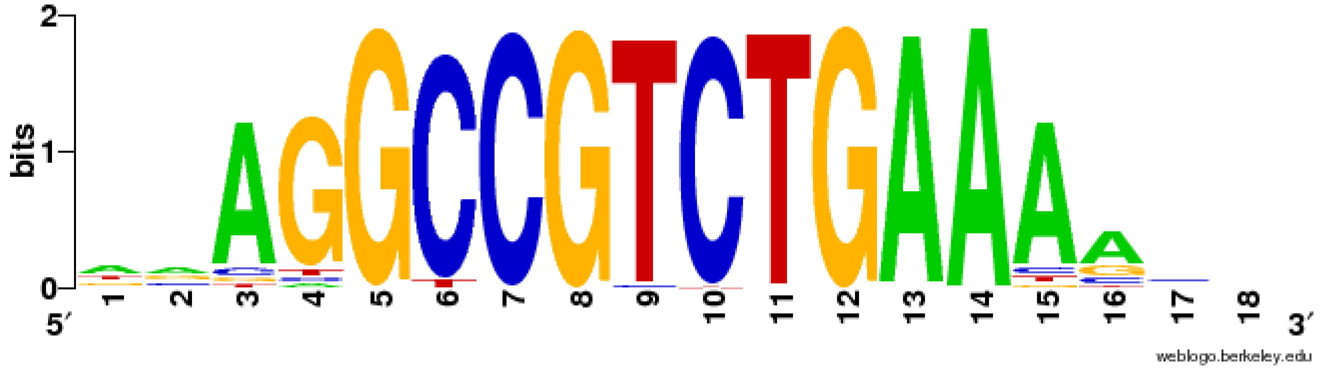

Neisseria sp. oral taxon 014 str.F0314 (AG-DUS)

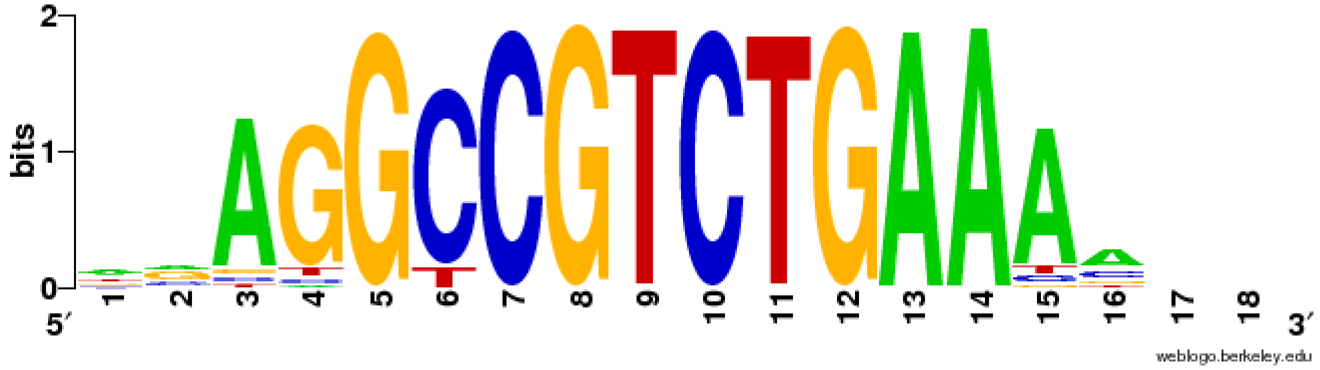

Neisseria mucosa ATCC 25996 (AG-mucDUS)

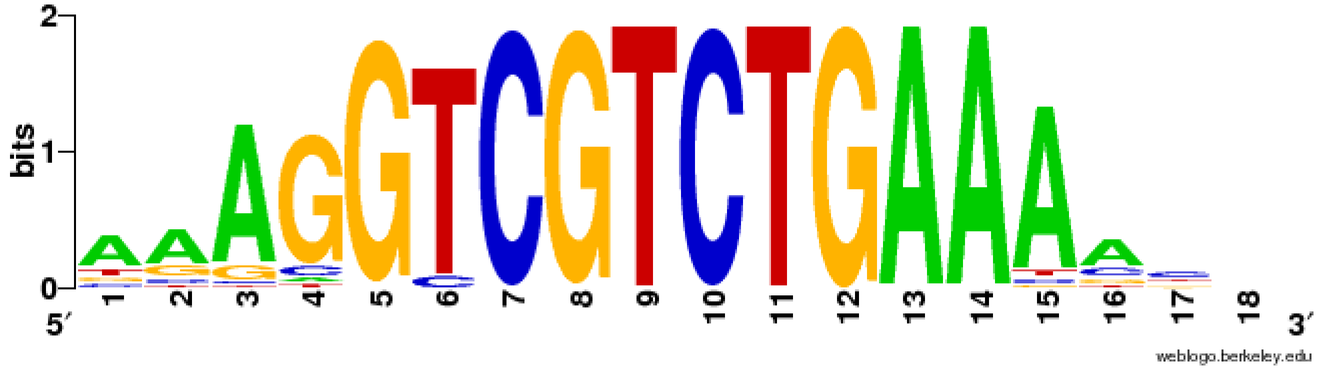

Figure S1: Sequence logos, single divergence permitted

D

Eikenella corrodens ATCC 23834 (AG-eikDUS)

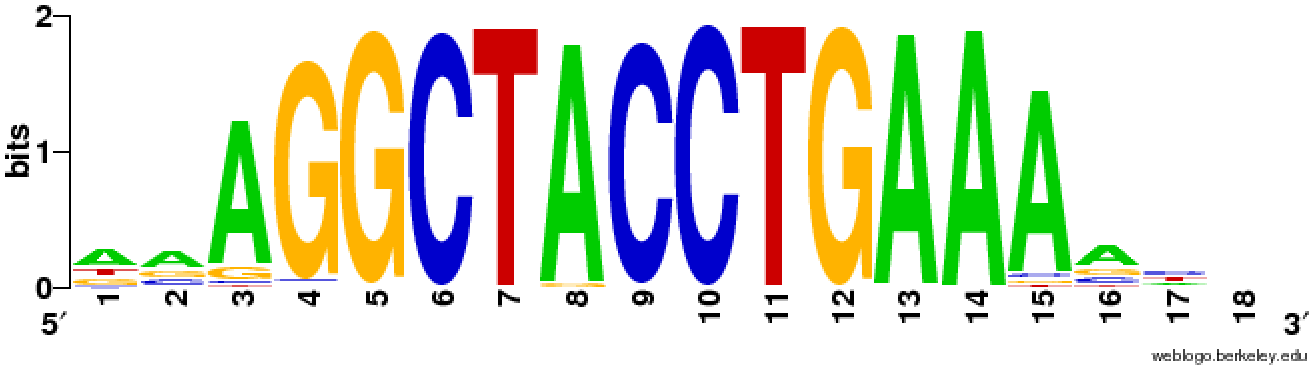

Kingella oralis ATCC 51147 (AG-kingDUS)

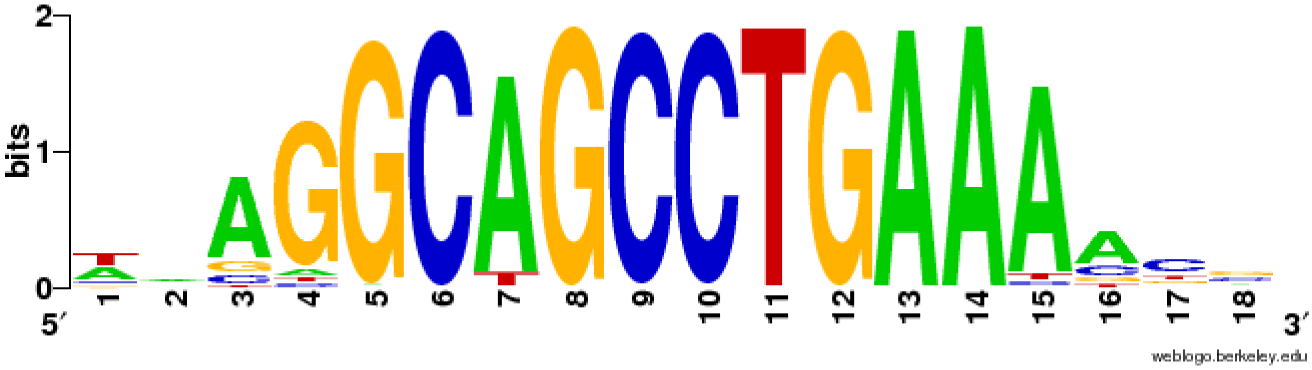

Kingella denitrificans ATCC 33394 (AA-king3DUS)

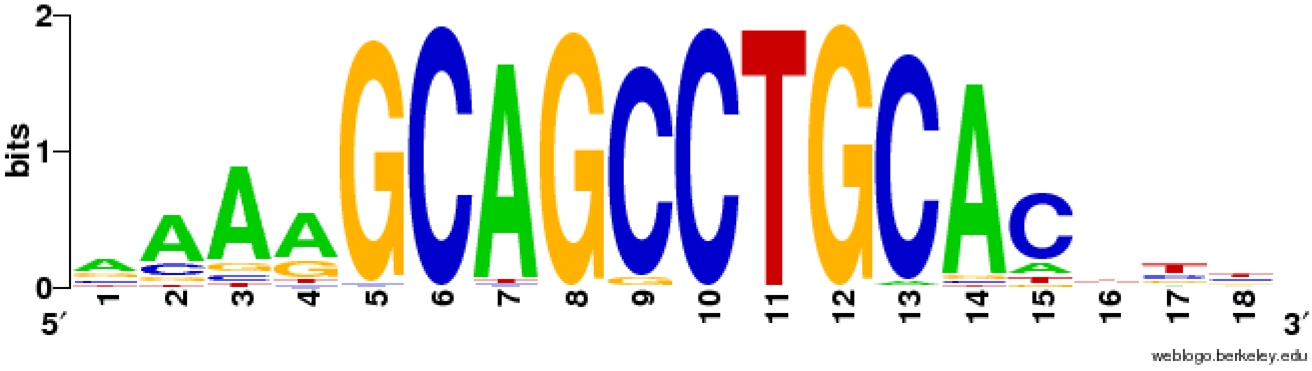

Figure S1: Sequence logos, single divergence permitted

E

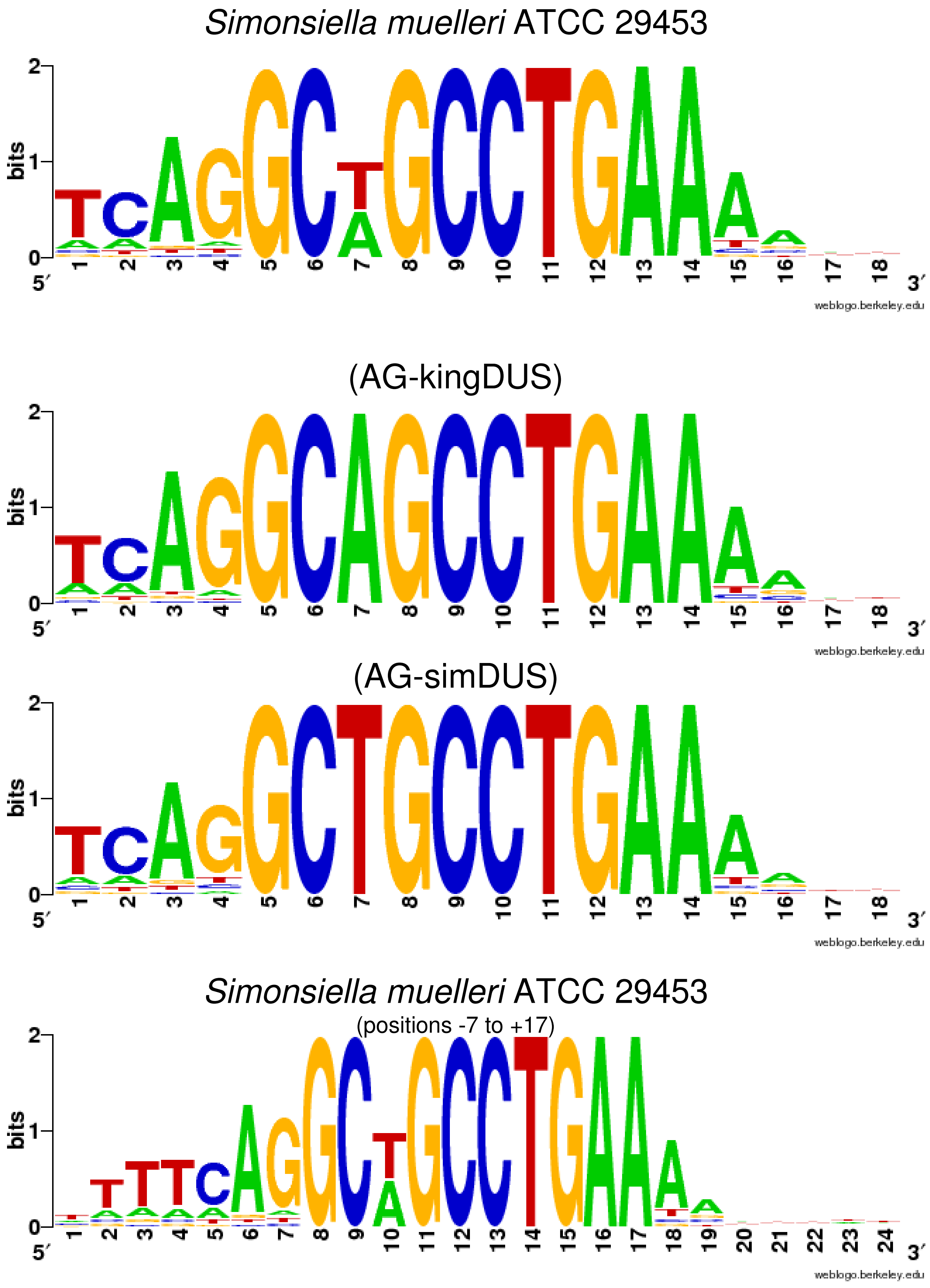

Figure S1: Sequence logos, no divergence permitted
