# Supplementary material for: Dialects of the DNA Uptake Sequence in Neisseriaceae
Source: PLoS Genet. 2013 Apr 18;9(4):e1003458. doi: 10.1371/journal.pgen.1003458 (PMC3630211; doi:10.1371/journal.pgen.1003458)
Supplement: Figure S2 — Quantitative transformations of N. meningitidis MC58 with DUS containing point mutations. The graphs show the transformation frequencies used for Figure 3A. The variations between the seven experiments in the range are visible as well as the relative consistent relation to the internal standard AT-DUS. (PDF) [file pgen.1003458.s002.pdf]

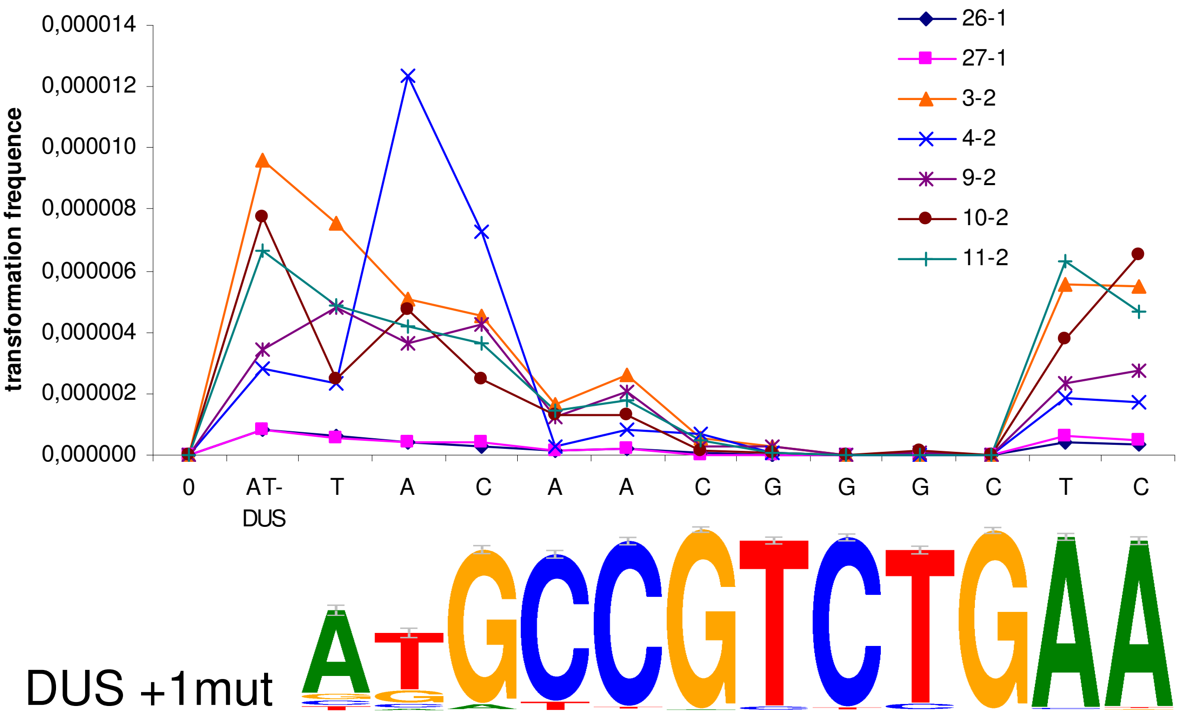

Figure S2: *Neisseria menigitidis* MC58 transformation results from seven independent experiments
