# Supplementary material for: Dialects of the DNA Uptake Sequence in Neisseriaceae
Source: PLoS Genet. 2013 Apr 18;9(4):e1003458. doi: 10.1371/journal.pgen.1003458 (PMC3630211; doi:10.1371/journal.pgen.1003458)
Supplement: Figure S3 — Quantitative transformations of N. meningitidis MC58 with DUS variants. The graphs show the transformation frequencies from seven independent experiments which were used for Figure 4A. Range variations with consistent ranking are seen as in Figure S2. (PDF) [file pgen.1003458.s003.pdf]

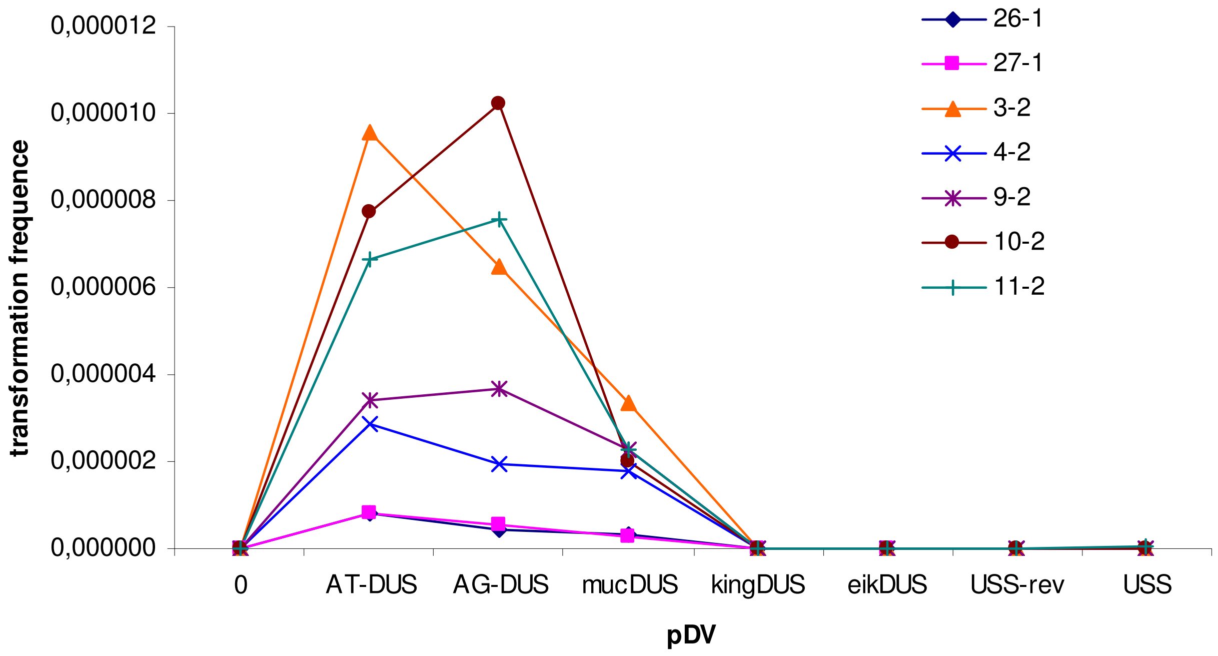

Figure S3: *Neisseria menigitidis* MC58 transformation results from seven independent experiments
