# Supplementary figures and images for: Dialects of the DNA Uptake Sequence in Neisseriaceae
Source: PLoS Genet. 2013 Apr 18;9(4):e1003458. doi: 10.1371/journal.pgen.1003458 (PMC3630211; doi:10.1371/journal.pgen.1003458)

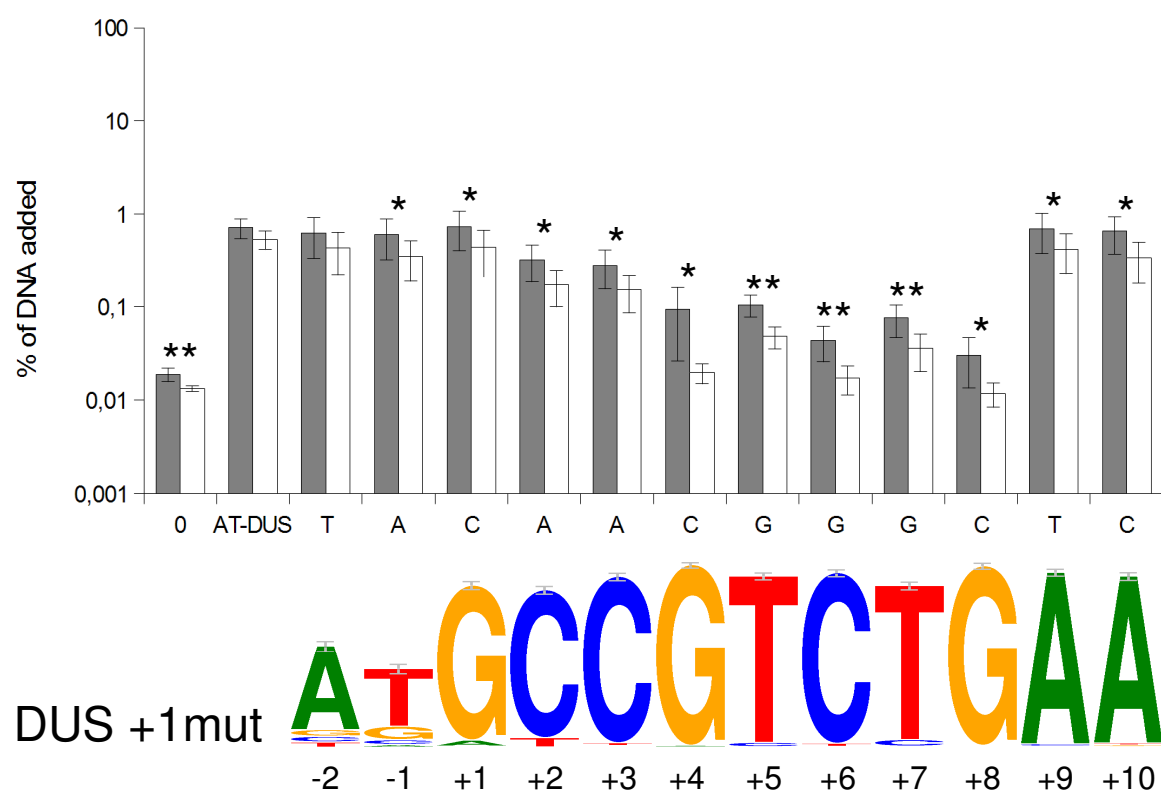

Figure S4: *Neisseria meningitidis* 8013 DNA binding and uptake

Supplement: Figure S4 — Effects of point mutations in the DUS on DNA binding and uptake of N. meningitidis 8013. Equivalent to Figure 3B. Data derived with the N. meningitidis strain 8013. Quantification of the binding of radiolabelled DNA to live cells. Total DNA binding shown as gray bars and benzonase resistant DNA (uptake) shown as white bars plotted as percentage of DNA added. Average values from 4 independent experiments are shown and standard deviations are indicated by bars. Student's t-test results for DNA binding versus uptake are marked by stars (p≤0.2 = *, p≤0.05 = **, p≤0.001 = ***). DUS sequence transversions are given as abscissa labels. Sequence logo as in Figure 3. (PDF) [file pgen.1003458.s004.pdf]

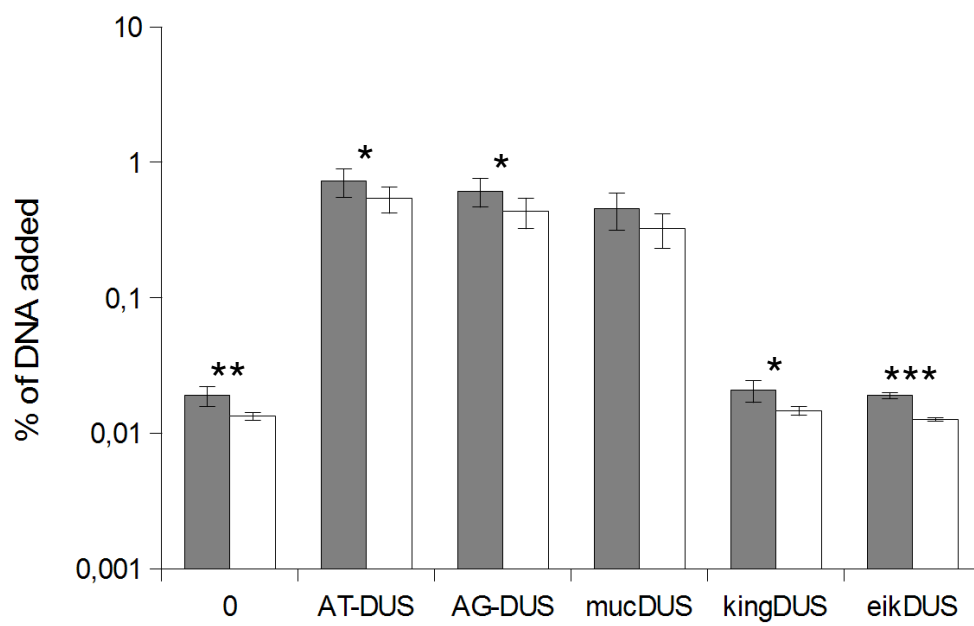

Figure S5: *Neisseria meningitidis* 8013 DNA binding and uptake

Supplement: Figure S5 — Quantification of DNA binding and uptake of N. meningitidis 8013 with DUS from other Neisseriaceae. Equivalent to Figure 4B. Data derived with the N. meningitidis strain 8013. Total DNA binding shown as gray bars and benzonase resistant DNA (uptake) shown as white bars plotted as percentage of DNA added. Results form 3 independent experiments are represented. Abscissa labels give the DUS variant. Student's t-test values are indicated (p≤0.2 = *, p≤0.05 = **, p≤0.001 = ***). (PDF) [file pgen.1003458.s005.pdf]

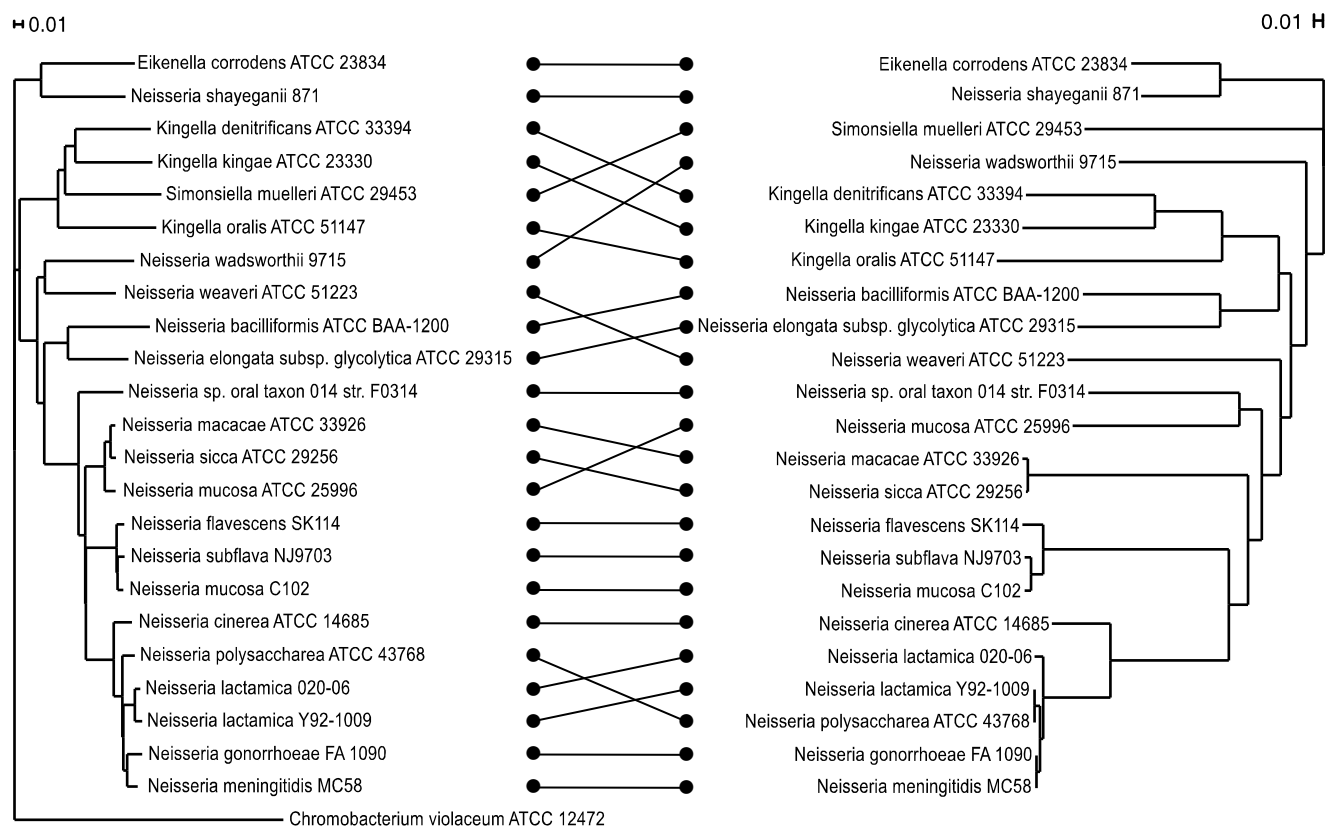

Figure S7: Comparison of the core genome based and the ComP based phylogeny

Supplement: Figure S7 — Comparison of the core genome based and the ComP based phylogeny. The phylogenetic tree from Figure 2 (left) is compared to the ComP based tree (right) with connectors showing the relations. Homologues to N. meningitidis strain MC58 ComP were identified with EDGAR [67] and BLAST [71] and the ComP phylogram was based on a ClustalW generated alignment of the globular domain (residues 35–149) of ComP. The scale bars represent 0.01 substitutions per nucleotide site. (PDF) [file pgen.1003458.s007.pdf]
