# Supplementary material for: Dialects of the DNA Uptake Sequence in Neisseriaceae
Source: PLoS Genet. 2013 Apr 18;9(4):e1003458. doi: 10.1371/journal.pgen.1003458 (PMC3630211; doi:10.1371/journal.pgen.1003458)
Supplement: Figure S6 — Dyad symmetry structures. Graphical representation of the DUS containing dyad symmetry structures found in the genomes of Simonsiella muelleri ATCC 29453 (A) and Kingella denitrificans ATCC 33394 (B) and the number of their occurrences in selected genomes. The translations of the three coding frames are given on top with the variable positions shaded in gray. (PDF) [file pgen.1003458.s006.pdf]

# A

```
               Q  A  A  *
              S  G  S  L
             F  R  Q  P  E
             TTCAGGCAGCCTGAA  AG-kingDUS
      simDUS AAGTCCGTCGGACTT
   (reverse)
```

```
               Q  A  A  *
              S  G  C  L
             F  R  L  P  E
             TTCAGGCTGCCTGAA  AG-simDUS
  AG-kingDUS AAGTCCGACGGACTT
   (reverse)
```

| | |
|---|---|
| *Simonsiella muelleri* ATCC 29453: | n=1294 |
| *Kingella oralis* ATCC 51147: | n= 318 |
| *Kingella kingae* ATCC 23330: | n= 161 |
| *Kingella denitrificans* ATCC 33394: | n= 30 |
| *Eikenella corrodens* ATCC 23834: | n= 5 |
| *Neisseria meningitidis* MC58: | n= 7 |

# B

```
               Q  A  A
              A  G  C
             C  R  L
             TGCAGGCTGCT  T-king2DUS
  A-king3DUS ACGTCCGACGA
   (reverse)
```

```
               Q  P  A
              A  A  C
             S  S  L
             AGCAGCCTGCA  A-king3DUS
  T-king2DUS TCGTCGGACGT
   (reverse)
```

| | |
|---|---|
| *Kingella denitrificans* ATCC 33394: | n=1405 |
| *Kingella kingae* ATCC 23330: | n=1217 |
| *Eikenella corrodens* ATCC 23834: | n= 44 |
| *Kingella oralis* ATCC 51147: | n= 8 |
| *Neisseria meningitidis* MC58: | n= 8 |
| *Simonsiella muelleri* ATCC 29453: | n= 5 |

Figure S6: Dyad symmetry structures based on DUS sequences, marked with black background; kingDUS and simDUS (A); king3DUS and king2DUS (B). In the translations of the three reading frames variable positions are shown with a gray background. The number of occurences of this structures in selected genomes are given. The king2DUS was not considered a separate DUS dialect as there are only 77 king2DUS in the genome of *K. denitrificans* that are not occurring together with king3DUS.
