# Supplementary material for: Dialects of the DNA Uptake Sequence in Neisseriaceae
Source: PLoS Genet. 2013 Apr 18;9(4):e1003458. doi: 10.1371/journal.pgen.1003458 (PMC3630211; doi:10.1371/journal.pgen.1003458)
Supplement: Table S1 — Number of DUS sequences in Neisseriaceae. Counts of DUS in the genomes of species that contain A) the canonical DUS, B) the mucDUS, and C) other DUS variants, and D) other over-represented sequences in other Neisseriaceae. The overrepresented sequences were detected with the repeat-finder Perl script [21] and counts obtained using fuzznuc [60]. (PDF) [file pgen.1003458.s008.pdf]

Table S1: Number of over-represented sequences in *Neisseriaceae*

A) Canonical DUS

strain	DUS	DUS +1mut [1]	mucDUS
Neisseria (with AT-DUS)			
Neisseria meningitidis			
053442	1858	2678	151
8013	1915	2729	148
961-5945	1926	2728	180
ATCC 13091	1925	2717	157
CU385	1921	2726	170
ES14902	1882	2696	161
FAM18	1888	2704	166
G2136	1889	2702	168
H44/76	1911	2713	160
M01-240149	1935	2706	149
M01-240355	1906	2758	171
M04-240196	1928	2738	169
M0579	1958	2743	150
M13399	1928	2732	163
M6190	1892	2706	160
MC58	1935	2742	165
N1568	1913	2743	157
NZ-05/33	1947	2721	149
OX99.30304	1843	2600	155
WUE2594	1933	2769	166
Z2491	1892	2705	168
α14	1851	2701	180
α153	1831	2599	153
α275	1862	2672	160
α710	1939	2714	150
Neisseria gonorrhoeae			
1291	1897	2652	103
35/02	1917	2668	116
DGI18	1920	2656	107
DGI2	1928	2672	109
F19	1932	2690	109
F6140	1919	2668	110
F62	1967	2739	118
FA1090	1965	2734	118
MS11	1919	2685	108
NCCP11945	1966	2766	129
PID1	1927	2688	115

[1] DUS+1mut = number of DUS plus the number of DUS sequences with one mutation

strain	DUS	DUS +1mut [1]	mucDUS
Neisseria (with AT-DUS)			
PID18	1918	2683	111
PID24-1	1922	2662	107
PID332	1940	2701	111
SK-92-1035	1913	2644	107
SK-92-679	1912	2671	109
Neisseria lactamica			
ATCC 23970	2213	2820	111
NS19	2102	2708	121
ST640	2245	2807	91
Y92-1009	2113	2620	82
Neisseria cinerea			
ATCC 14685	943	1372	113
Neisseria polysaccharea			
ATCC 43768	2183	2831	138
Neisseria (with AG-DUS)			
Neisseria sp. oral taxon 014			
str. F0314	3236	4069	467
Neisseria subflava			
NJ9703	2871	3511	166
Neisseria flavescens			
NRL30031 H210	1196	1585	86
SK114	2767	3413	247
Neisseria mucosa			
C102	2964	3466	155
Neisseria bacilliformis			
ATCC BAA-1200	4265	4914	140
Neisseria weaveri			
ATCC 51223	2842	3355	15
LMG 5135	2875	3415	15
Neisseria elongata			
subsp. glycolytica ATCC 29315	3273	3885	174

B) mucDUS

strain	DUS	DUS +1mut	mucDUS	mucDUS +1mut
Neisseria mucosa				
ATCC 25996	179	1988	1543	1934
Neisseria sicca				
NRL 30,016	298	4452	3770	4378
DS1	229	3798	3212	3827
4320	212	3852	3294	3893
Neisseria macacae				
ATCC 33926	300	4362	3729	4299
Neisseria sp.				
Neisseria sp. GT4A CT1	270	4286	3670	4227

C) Other DUS

strain	DUS	DUS +1mut		
Neisseria wadsworthii			**wadDUS**	**wadDUS +1mut**
9715	381	679	2426	2833
Kingella oralis			**kingDUS**	**kingDUS +1mut**
ATCC 51147	76	320	5918	7153
Simonsiella muelleri			**kingDUS**	**kingDUS +1mut**
ATCC 29453	14	204	2257	5345
			**simDUS**	**simDUS +1mut**
			2292	5342
Kingella kingae			**king3DUS**	**king3DUS +1mut**
ATCC 23330	12	142	2787	3678
269-492	no counts available [1]			
Kingella denitrificans			**king3DUS**	**king3DUS +1mut**
ATCC 33394	79	371	3603	4982
Eikenella corrodens			**eikDUS**	**eikDUS +1mut**
ATCC 23834	112	326	3269	3706
Neisseria shayeganii			**eikDUS**	**eikDUS +1mut**
871	82	301	2245	2568

[1] = king3DUS was found in *K. kingae* strain 269-492 based on the analysis of the following sequences obtained from GenBank: gi209864116, gi209864114; gi193734239, gi193734232, gi193734228, gi117956037, gi193734244, gi193734236, gi193734229. A total of 32 king3DUS were found in 47kb of sequence.

D) Over-represented 10-mer sequences in the genomes of other *Neisseriaceae*

strain	sequence	counts
Laribacter hongkongensis		
HLHK9	GCCGGCCAGC [1]	485
	CATGAAAAAA [2]	48
Lutiella nitroferrum		
2002	GTTGGCCGAA [1, 2]	1104
Pseudogulbenkiania		
sp. NH8B	GTTGGCCGAA [1, 2]	1153
Chromobacterium		
sp. C-61	CGCCGCCGGC [1]	2242 [3]
	AGATCGTAAA [2]	352 [3]
Chromobacterium violaceum		
ATCC 12472	CAGCAGCAGC [1]	714
	AATAAAAAAA [2]	23

[1] = highest number of counts
[2] = most over-represented sequence
[3] = based on the cumulated 1119 contigs
