# Supplementary material for: Dialects of the DNA Uptake Sequence in Neisseriaceae
Source: PLoS Genet. 2013 Apr 18;9(4):e1003458. doi: 10.1371/journal.pgen.1003458 (PMC3630211; doi:10.1371/journal.pgen.1003458)
Supplement: Table S2 — Inverted DUS repeats in Neisseriaceae. Counts of DUS containing inverted repeats in the genomes of selected Neisseriaceae species. (PDF) [file pgen.1003458.s009.pdf]

Table S2: inverted DUS repeats in *Neisseriaceae*

bacterium	fuzznuc[1] search pattern	counts	counts (2 mut[1])
Eikenella corrodens ATCC 23834	GCTACCTGAA-N(1,19)-TTCAGGTAGC	431	529
Kingella denitrificans ATCC 33394	GCAGCCTGCA-N(1,19)-TGCAGGCTGC	401	554
Kingella oralis ATCC 51147	GCAGCCTGAA-N(1,19)-TTCAGGCTGC	550	686
Neisseria wadsworthii 9715	CCTGTCTGAA-N(1,19)-TTCAGACAGG	582	683
Neisseria elongata subsp. *glycolytica* ATCC 29315	GCCGTCTGAA-N(1,19)-TTCAGACGGC	487	598
Neisseria mucosa ATCC 25996	GTCGTCTGAA-N(1,19)-TTCAGACGAC	674	776
Neisseria flavescens SK114	GCCGTCTGAA-N(1,19)-TTCAGACGGC	556	675
Neisseria meningitidis MC58	GCCGTCTGAA-N(1,19)-TTCAGACGGC	380	555

[1] = part of the EMBOSS package [1]

[2] = two mutations in the search pattern allowed

Reference:

1. Rice P, Longden I, Bleasby A (2000) EMBOSS: the European Molecular Biology Open Software Suite. Trends Genet 16: 276-277.
