# Supplementary material for: Dialects of the DNA Uptake Sequence in Neisseriaceae
Source: PLoS Genet. 2013 Apr 18;9(4):e1003458. doi: 10.1371/journal.pgen.1003458 (PMC3630211; doi:10.1371/journal.pgen.1003458)
Supplement: Table S3 — DUS variation oligonucleotides. Primers employed in the PCR based amplification of the pilG::ermC fragment from the plasmid p0-DUS. (PDF) [file pgen.1003458.s010.pdf]

Table S3: DUS variation oligonucleotides

primer name	RS[1]	sequence 3'→ 5' [2]
OH3	*Sac*II	TAGACCGCGGTCAGGCGACCACGTTGCC
OH11_DUS	*Xho*I	ACGACTCGAGAT**GCCGTCTGAA**ATGGCTAAAAACGGAGGAT
9636OHA11_DUS_B	*Xho*I	ACGACTCGAGTT**GCCGTCTGAA**ATGGCTAAAAACGGAGGAT
9637OHA11_DUS_C	*Xho*I	ACGACTCGAGAA**GCCGTCTGAA**ATGGCTAAAAACGGAGGAT
9638OHA11_DUS_D	*Xho*I	ACGACTCGAGAT**CCCGTCTGAA**ATGGCTAAAAACGGAGGAT
9639OHA11_DUS_E	*Xho*I	ACGACTCGAGAT**GACGTCTGAA**ATGGCTAAAAACGGAGGAT
9640OHA11_DUS_F	*Xho*I	ACGACTCGAGAT**GCAGTCTGAA**ATGGCTAAAAACGGAGGAT
9641OHA11_DUS_G	*Xho*I	ACGACTCGAGAT**GCCCTCTGAA**ATGGCTAAAAACGGAGGAT
9642OHA11_DUS_H	*Xho*I	ACGACTCGAGAT**GCCGGCTGAA**ATGGCTAAAAACGGAGGAT
9643OHA11_DUS_I	*Xho*I	ACGACTCGAGAT**GCCGTGTGAA**ATGGCTAAAAACGGAGGAT
9644OHA11_DUS_J	*Xho*I	ACGACTCGAGAT**GCCGTCGGAA**ATGGCTAAAAACGGAGGAT
9645OHA11_DUS_K	*Xho*I	ACGACTCGAGAT**GCCGTCTCAA**ATGGCTAAAAACGGAGGAT
9646OHA11_DUS_L	*Xho*I	ACGACTCGAGAT**GCCGTCTGTA**ATGGCTAAAAACGGAGGAT
9647OHA11_DUS_M	*Xho*I	ACGACTCGAGAT**GCCGTCTGAC**ATGGCTAAAAACGGAGGAT
9648OHA11_DUS_N	*Xho*I	ACGACTCGAGAG**GCCGTCTGAA**ATGGCTAAAAACGGAGGAT
9649OHA11_DUS_O (mucDUS)	*Xho*I	ACGACTCGAGAG**GTCGTCTGAA**ATGGCTAAAAACGGAGGAT
9650OHA11_DUS_P (kingDUS)	*Xho*I	ACGACTCGAGAG**GCAGCCTGAA**ATGGCTAAAAACGGAGGAT
9651OHA11_DUS_Q (eikDUS)	*Xho*I	ACGACTCGAGAG**GCTACCTGAA**ATGGCTAAAAACGGAGGAT
OHA2025_USS_Hinfrev	*Xho*I	ACGACTCGAG**TGGCGTGAA**ATGGCTAAAAACGGAGGAT
OHA2024_USS_Hinf	*Xho*I	ACGACTCGAGA**AAGTGCGGT**TGGCTAAAAACGGAGGAT

[1] = restriction site

[2] = DUS and mutated DUS sequences in bold
