# Supplementary material for: Dialects of the DNA Uptake Sequence in Neisseriaceae
Source: PLoS Genet. 2013 Apr 18;9(4):e1003458. doi: 10.1371/journal.pgen.1003458 (PMC3630211; doi:10.1371/journal.pgen.1003458)
Supplement: Table S4 — Plasmids and bacteria. Bacterial strains and plasmids employed in this study. (PDF) [file pgen.1003458.s011.pdf]

Table S4: Bacteria and plasmids

name	relevant characteristics	reference
Escherichia coli ER2566	F^- λ- *fhuA2* [*lon*] *ompT lacZ*::T7 gene1 *gal sulA11* Δ(*mcrC-mrr*)114::IS10 R(*mcr-73*::miniTn10-Tet^S)2 R(*zgb-210*::Tn10)(Tet^S) *endA1* [*dcm*]	New England Biolabs
Escherichia coli XL-1 Blue	*recA1 endA1 gyrA96 thi-1 hsdR17 supE44 relA1 lac* [F' *proAB* $lacI^qZ\Delta M15$ *Tn10* (Tet^r)]	Stratagene
Neisseria meningitidis strain MC58	serogroup B	[1]
Neisseria meningitidis strain 8013	serogroup C	[2]
Neisseria elongata<br>subsp. glycolytica 6171/75<br>(= ATCC 29315 = NCTC 11050<br>= DSM 23337)	type strain	[3]
Neisseria mucosa<br>(= ATCC 19696 = NCTC 12978<br>= DSM 17611)	type strain	[4]
Neisseria mucosa<br>(= ATCC 25996 = NCTC 10774<br>= DSM 4631)	isolated from human pharyngeal mucosa	[5,6]
Neisseria sicca NRL 30,016<br>(= ATCC 29259 = DSM 17713)	type strain	[7]
Kingella oralis<br>(ATCC 51147 = DSM 18271)	type strain	[8]
Kingella denitrificans<br>(= ATCC 33394 = NCTC 10995<br>= DSM 10202)	type strain	[9]
Eikenella corrodens strain 31745	isolated from spinal fluid	[10]
Eikenella corrodens<br>(ATCC 23834 = NCTC 10596<br>= DSM 8340)	type strain	[11]
p0-DUS	pBluescript II SK+<br>harbouring *pilG*::mTnErm	[12]
pOHA-D4	AT-DUS	this study
pSAF-DV-36	TT-DUS	this study
pOHA-DV-37	AA-DUS	this study
pSAF-DV-38	AT-**C**CCGTCTGAA	this study
pSAF-DV-39	AT-G**A**CGTCTGAA	this study
pSAF-DV-40	AT-GC**A**GTCTGAA	this study
pSAF-DV-41	AT-GCC**C**TCTGAA	this study
pSAF-DV-42	AT-GCCG**G**CTGAA	this study
pOHA-DV-43	AT-GCCGT**G**TGAA	this study
pSAF-DV-44	AT-GCCGTC**G**GAA	this study
pSAF-DV-45	AT-GCCGTCT**C**AA	this study
pSAF-DV-46	AT-GCCGTCTG**T**A	this study
pOHA-DV-47	AT-GCCGTCTGA**C**	this study
pSAF-DV-48	AG-DUS	this study
pSAF-DV-49	AG-mucDUS	this study
pSAF-DV-50	AG-kingDUS	this study
pSAF-DV-51	AG-eikDUS	this study
pSAF-DV-52	reverse USS	this study
pSAF-DV-53	USS	this study

References

1. Tettelin H, Saunders NJ, Heidelberg J, Jeffries AC, Nelson KE, et al. (2000) Complete genome sequence of *Neisseria meningitidis* serogroup B strain MC58. Science 287: 1809-1815.
2. Nassif X, Lowy J, Stenberg P, O'Gaora P, Ganji A, et al. (1993) Antigenic variation of pilin regulates adhesion of Neisseria meningitidis to human epithelial cells. Mol Microbiol 8: 719-725.
3. Henriksen SD, Holten E (1976) *Neisseria elongata* subsp. *glycolytica* subsp. nov. International Journal of Systematic Bacteriology October 1976: 478-481.
4. Veron M, Thibault P, Second L (1959) [*Neisseria mucosa* (*Diplococcus mucosus* Lingelsheim). I. Bacteriological description and study of its pathogenicity]. Ann Inst Pasteur (Paris) 97: 497-510.
5. Berger U (1971) [Neisseria mucosa var. heidelbergensis]. Z Med Mikrobiol Immunol 156: 154-158.
6. Roberts RJ (1985) Restriction and modification enzymes and their recognition sequences. Nucleic Acids Res 13 Suppl: r165-200.
7. Bøvre K (1984) Family VIII. *Neisseriaceae*. In: Krieg NR, Holt JG, editors. Bergey's manual of systematic bacteriology. Baltimore: Williams & Wilkins. pp. 288-309.
8. Dewhirst FE, Chen CK, Paster BJ, Zambon JJ (1993) Phylogeny of species in the family Neisseriaceae isolated from human dental plaque and description of Kingella oralis sp. nov [corrected]. Int J Syst Bacteriol 43: 490-499.
9. Weir S, Lee LW, Marrs CF (1996) Identification of four complete type 4 pilin genes in a single *Kingella denitrificans* genome. Infect Immun 64: 4993-4999.
10. Tønjum T, Hagen N, Bøvre K (1985) Identification of *Eikenella corrodens* and *Cardiobacterium hominis* by genetic transformation. Acta Pathol Microbiol Immunol Scand B 93: 389-394.
11. Henriksen SD (1969) Corroding bacteria from the respiratory tract. 2. *Bacteroides corrodens*. Acta Pathol Microbiol Scand 75: 91-96.
12. Ambur OH, Frye SA, Tønjum T (2007) New functional identity for the DNA uptake sequence in transformation and its presence in transcriptional terminators. J Bacteriol 189: 2077-2085.
