# Supplementary material for: Dialects of the DNA Uptake Sequence in Neisseriaceae
Source: PLoS Genet. 2013 Apr 18;9(4):e1003458. doi: 10.1371/journal.pgen.1003458 (PMC3630211; doi:10.1371/journal.pgen.1003458)
Supplement: Table S5 — RpsL specific PCR primer. Primers employed for the PCR-based amplification of the rpsL gene. (PDF) [file pgen.1003458.s012.pdf]

Table S5: *RpsL* specific PCR primer

target	primer name	orientation	DUS	RS[1]	sequence 3'→ 5' [2]
Kingella denitrificans	OHA2235	forward	AA-king3DUS	*Sac*I	GTGAGCTCAA**GCAGCCTGCA**CATGCCAACGATTAACCAATTGGTTCGCA
	OHA2236	forward	AT-DUS	*Sac*I	GTGAGCTCAT**GCCGTCTGAA**AATGCCAACGATTAACCAATTGGTTCGCA
	OHA2239	reverse	-	*Xho*I	GTCTCGAGTTATTTAGGGCGTTTAGCACCGTATTTAGAA
Eikenella corrodens	OHA2146	forward	AG-eikDUS	*Sac*I	GTGAGCTCT**TTCAGGTAGCCT**ATGCCAACTATTAACCAGTTAGTACGCA
	OHA2148	reverse	AG-eikDUS	*Xho*I	GTCTCGAG**AGGCTACCTGAA**ATTATTTCGGGCGTTTAGCACCGTATT
	OHA2147	forward	AT-DUS	*Sac*I	GTGAGCTCT**TTCAGACGGC**ATATGCCAACTATTAACCAGTTAGTACGCA
	OHA2149	reverse	AT-DUS	*Xho*I	GTCTCGAGAT**GCCGTCTGAA**ATTATTTCGGGCGTTTAGCACCGTATT
Neisseria elongata subsp. *glycolytica*	OHA2137	forward	AT-DUS	*Sac*I	GTGAGCTC**TTCAGACGGC**ATATGCCAACTATCAACCAATTGGTACGCA
	SF137	reverse	AT-DUS	*Xho*I	GTCTCGAG**TTCAGACGGC**ATTTATTTAGGACGCTTAGCACCGTATT
	SF138	forward	AG-DUS	*Sac*I	GTGAGCTC**TTCAGACGGC**CTATGCCAACTATCAACCAATTGGTACGCA
	SF139	reverse	AG-DUS	*Xho*I	GTCTCGAG**TTCAGACGGC**CTTTATTTAGGACGCTTAGCACCGTATT
	OHA2136	forward	AG-mucDUS	*Sac*I	GTGAGCTC**TTCAGACGAC**CTATGCCAACTATCAACCAATTGGTACGCA
	SF140	reverse	AG-mucDUS	*Xho*I	GTCTCGAG**TTCAGACGAC**CTTTATTTAGGACGCTTAGCACCGTATT

[1] = restriction site
[2] = DUS sequences in bold
